# A time-causal and time-recursive scale-covariant scale-space representation of temporal signals and past time

**DOI:** 10.1007/s00422-022-00953-6

**Published:** 2023-01-23

**Authors:** Tony Lindeberg

**Affiliations:** Computational Brain Science Lab, Division of Computational Science and Technology, KTH Royal Institute of Technology, 100 44 Stockholm, Sweden

**Keywords:** Time, Temporal, Scale, Time-causal, Time-recursive, Scale covariance, Scale space, Wavelet analysis, Time-frequency analysis, Signal, The present, Delay, Memory, Perceptual agent, Theoretical neuroscience, Theoretical biology

## Abstract

This article presents an overview of a theory for performing temporal smoothing on temporal signals in such a way that: (i) temporally smoothed signals at coarser temporal scales are guaranteed to constitute simplifications of corresponding temporally smoothed signals at any finer temporal scale (including the original signal) and (ii) the temporal smoothing process is both time-causal and time-recursive, in the sense that it does not require access to future information and can be performed with no other temporal memory buffer of the past than the resulting smoothed temporal scale-space representations themselves. For specific subsets of parameter settings for the classes of linear and shift-invariant temporal smoothing operators that obey this property, it is shown how temporal scale covariance can be additionally obtained, guaranteeing that if the temporal input signal is rescaled by a uniform temporal scaling factor, then also the resulting temporal scale-space representations of the rescaled temporal signal will constitute mere rescalings of the temporal scale-space representations of the original input signal, complemented by a shift along the temporal scale dimension. The resulting time-causal limit kernel that obeys this property constitutes a canonical temporal kernel for processing temporal signals in real-time scenarios when the regular Gaussian kernel cannot be used, because of its non-causal access to information from the future, and we cannot additionally require the temporal smoothing process to comprise a complementary memory of the past beyond the information contained in the temporal smoothing process itself, which in this way also serves as a multi-scale temporal memory of the past. We describe how the time-causal limit kernel relates to previously used temporal models, such as Koenderink’s scale-time kernels and the ex-Gaussian kernel. We do also give an overview of how the time-causal limit kernel can be used for modelling the temporal processing in models for spatio-temporal and spectro-temporal receptive fields, and how it more generally has a high potential for modelling neural temporal response functions in a purely time-causal and time-recursive way, that can also handle phenomena at multiple temporal scales in a theoretically well-founded manner. We detail how this theory can be efficiently implemented for discrete data, in terms of a set of recursive filters coupled in cascade. Hence, the theory is generally applicable for both: (i) modelling continuous temporal phenomena over multiple temporal scales and (ii) digital processing of measured temporal signals in real time. We conclude by stating implications of the theory for modelling temporal phenomena in biological, perceptual, neural and memory processes by mathematical models, as well as implications regarding the philosophy of time and perceptual agents. Specifically, we propose that for A-type theories of time, as well as for perceptual agents, the notion of a non-infinitesimal inner temporal scale of the temporal receptive fields has to be included in representations of the present, where the inherent nonzero temporal delay of such time-causal receptive fields implies a need for incorporating predictions from the actual time-delayed present in the layers of a perceptual hierarchy, to make it possible for a representation of the perceptual present to constitute a representation of the environment with timing properties closer to the actual present.

## Introduction

When processing time-dependent measurement signals, there is often a need to perform temporal smoothing prior to more refined data analysis. A commonly stated general motivation for this need is to suppress measurement noise, often based on the assumption that there is a well-defined underlying noise free signal that has been corrupted with some amount of measurement noise.

A more fundamental approach to take on the need for performing temporal smoothing of temporal signals is to follow a multi-scale approach, based on the observation that measurements performed on real-world data may reflect different types of temporal structures at different temporal scales. In other words, even for the underlying noise free signal in the above signal+noise model, it may hold that the data reflect different types of underlying physical or biological processes at different temporal scales. The measurement process itself, by which a non-infinitesimal amount of energy needs to be integrated over some non-infinitesimal temporal duration on the physical sensor, does in this respect define an inner temporal scale of the measurements, beyond which there is no way to resolve temporal phenomena that occur faster than this inner temporal scale. Any real-world physical measurement does in this respect involve an inherent notion of temporal scale.^1^

Specifically, in the areas of image processing, computer vision, machine listening^2^ and computational modelling of visual and auditory perception, this need is well understood, and has led to multi-scale approaches for spatial, spatio-temporal and spectro-temporal receptive fields expressed in terms of multi-scale representations over the spatial, spectral and temporal domains, where specifically the theoretical framework known as scale-space theory is based upon solid theory in terms of axiomatic derivations concerning how the multi-scale processing operations should be performed (Iijima 1962; Witkin 1983; Koenderink 1984; Koenderink and van Doorn 1987, 1992; Lindeberg 1993b, 1994, 2011, 2013b; Florack 1997; Sporring et al. 1997; Weickert et al. 1999; ter Haar Romeny 2003). It has also been found that biological perception, memory and cognition has developed biological processes at multiple temporal scales (DeAngelis et al. 1995; DeAngelis and Anzai 2004; Gütig and Sompolinsky 2006; Gentner 2008; Holcombe 2009; Goldman 2009; Gauthier et al. 2012; Atencio and Schreiner 2012; Chait et al. 2015; Teng et al. 2016; Buzsáki and Llinás 2017; Tsao et al. 2018; Osman et al. 2018; Latimer et al. 2019; Bright et al. 2020; Cavanagh et al. 2020; Monsa et al. 2020; Spitmaan et al. 2020; Howard and Hasselmo 2020; Howard 2021; Guo et al. 2021; Miri et al. 2022); see Sect. 7.3 for a more detailed retrospective review.

The subject of this article is to describe a theoretical framework for representing temporal signals at multiple temporal scales, intended for a more general audience without background in these areas and with the focus on the temporal domain only, thus without the complementary spatial or spectral domains that this theory has previously been combined with for expressing spatio-temporal and spectro-temporal receptive fields (Lindeberg and Fagerström 1996; Lindeberg 1997a, 2016, 2017, 2018a, b, 2021b; Lindeberg and Friberg 2015b, a). This theoretical framework, referred to as *temporal scale-space theory*, guarantees *non-creation of the temporal structures with increasing temporal scales,* in the sense that it ensures that a temporal representation at any coarser temporal scale constitutes a simplification of a temporal representation at any finer temporal scale, in the respect that the number of local temporal extrema, alternatively the number of temporal zero-crossings, is guaranteed to not increase from finer to coarser temporal scales.

Additionally, these temporal scale-space representations are *time-causal,* in the sense that they do not require access to future data, and are *time-recursive,* in the respect that the temporal representation at the next temporal moment can be computed with no other additional *memory of the past* than the temporal scale-space representation itself. For a specific choice of temporal scale-space kernel, referred to as the *time-causal limit kernel*, the temporal scale-space representations are also *scale covariant*, meaning that the set of temporal scale-space representations is closed under temporal rescalings of the input. A rescaling of the input signal by a uniform scaling factor merely corresponds to a rescaling of the temporal scale-space representations complemented by a shift of the temporal scale levels in the temporal scale-space representation. In this way, the temporal scale-space representation ensures an internally consistent way of processing temporal signals that may be subject to temporal scaling transformations, by phenomena or events that may occur faster or slower in the world.

A main purpose of this article is to describe this theory in a self-contained manner, without need for the reader to digest the original references, where the information is distributed over several papers, and may require a substantial effort for a reader not previously familiar with this framework, to get an updated view of the latest version of this theory.^3^ Furthermore, we will describe explicit relations to other previously used temporal models, such as Koenderink’s scale-time kernels (Koenderink 1988) and the ex-Gaussian model (Grushka 1972; Bright et al. 2020), making it possible to transfer modelling results from those temporal models to the time-causal limit kernel described in this article.

We will also relate the presented temporal scale-space theory to other approaches for processing signals at multiple temporal scales, such as wavelet analysis and time-frequency analysis. Specifically, we will outline how the temporal derivatives of the proposed time-causal limit kernel described and analyzed in this article allow for fully time-causal and time-recursive wavelet analysis methods, without need for additional temporal buffering, and thus enabling minimal temporal response times in a time-critical context. We will also outline how a complex-valued extension of the proposed time-causal limit kernel can be seen as a time-causal analogue of Gabor functions, thus allowing for capturing essentially similar transformations of temporal signals as for the family of Gabor functions, and thereby providing a way to define a scale-covariant time-frequency representation over a time-causal temporal domain, which by a slight modification can also be extended to additionally being implemented in terms strictly time-recursive operations.

Additionally, we will describe implications of using this theory for modelling perceptual, neural and memory processes in biological systems by mathematical models, as well as implications of the theory with regard to the philosophy of time and perceptual agents. Specifically, we will argue that when modelling a perceptual representation of the present, it is essential to include the inner temporal scales of the perceptual processes that lead to any percept, where the inherent temporal delays of such time-causal operations imply that a representation of the present will *de facto* constitute a representation of some temporal intervals in the past, unless complemented by prediction processes to enable better timing properties of a perceptual agent that interacts with a dynamic world.

### Structure of this article

This paper is organized as follows: Sect. 2 introduces the problem of constructing a temporal scale-space representation, as constituting a multi-scale representation of temporal signals, with the property that a measure of the amount of structure in the signal, quantified as the number of local extrema over time, must not increase from any finer to any coarser temporal scale. A complete classification of the time-causal convolution kernels that enable this property is given, and it is shown that the only possible time-causal scale-space kernels over a continuous temporal domain consist of truncated exponential kernels coupled in cascade.

Section 3 then adds a complementary condition on this structure, in terms of temporal scale covariance, and meaning that if the temporal input signal is rescaled by a uniform temporal scaling factor, then the result of temporal scale-space filtering of this kernel should also be a mere rescaling of the result of performing temporal scale-space filtering on the input signal, complemented by a shift in along the temporal scale axis and a possibly complementary shift in the magnitude of the signal. It is shown that a specific kernel, the time-causal limit kernel, defined from an infinite convolution of truncated exponential kernels in cascade, with specially chosen time constants, obeys temporal scale covariance. We do also show how this time-causal limit kernel relates to previously used temporal models, such as Koenderink’s scale-time kernels and the ex-Gaussian kernel.

In Sect. 4, we complement the above treatment for continuous signals with a corresponding discrete theory, ensuring that the number of local extrema in a discrete signal is also guaranteed to not increase from any finer to any coarser temporal scale. The discrete analogue of the truncated exponential kernels are first-order recursive filters coupled in cascade. Section 5 furthermore generalizes the above theory from temporal smoothing of a raw temporal signal, to the computation of temporal scale-space derivatives, which measure the amount of change in the signal with respect to any level of temporal scale. Section 6 outlines how the proposed temporal scale-space representation is related to other approaches for handling temporal signals at multiple temporal scales, specifically wavelet analysis and time-frequency analysis, with conceptual extensions of these notions with respect to strictly time-causal and time-recursive operations for real-time applications.

Section 7 describes how this general theory can be used for modelling time-dependent processes and mechanisms in perceptual and neural systems, with emphasis on spatio-temporal and spectro-temporal receptive fields as well as temporal memory processes. Section 8 outlines more general implications of the theory with regard to the philosophy of time and how time is handled by a perceptual agent. Specifically, we develop how the inner temporal scale associated with any biophysical measurement of time-dependent phenomena implies that a non-infinitesimal inner temporal scale needs to be included in a representation of the perceptual present, and also that the nonzero temporal delay of such time-causal kernels implies that a biophysical representation of the present will *de facto* constitute a representation of what has occurred over some temporal intervals in the past, in turn implying a need for prediction mechanisms to extrapolate the *de facto* time-delayed representation of the present into a better predicted representation of the actual present.

Section 9 gives a retrospective historic overview of the different parts of temporal scale-space theory that this paper is based on, follows and extends, as well as a conceptual overview of some of the main contributions to temporal scale-space theory made in this article. Finally, Sect. 10 summarizes some of the main results.

**Fig. 1:**
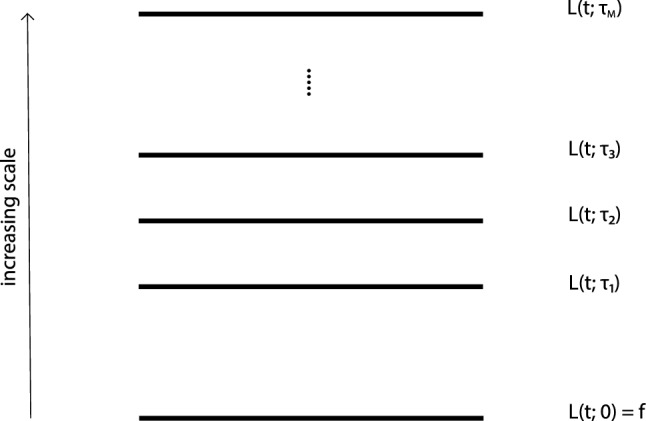
The main idea of a scale-space representation is to, given any input signal $f\mathrm{(t)}$, create a set of derived signals $L(t;\; \tau )$ intended to represent the information in the original signal at a set of coarser levels of scale $\tau$, with $L(t;\; 0) = f(t)$. These derived signals should preferably constitute true simplifications of each other, in such a way that the signal at a coarser level of scale does not contain more structures or information than any signal at any finer level of scale. Over spatial image domains, the notion of scale-space representation has been extensively studied, where several axiomatic derivations have shown that the Gaussian kernel and its corresponding Gaussian derivatives constitute a canonical class of convolution kernels for generating a spatial scale-space representation and have also been demonstrated to constitute a suitable basis of image primitives for computing different types of features from spatial image data. In this paper, we develop the associated notion of temporal scale-space theory, based on the additional constraints that (i) the temporal scale-space kernels are not allowed to access information from the future in relation to any time moment and that (ii) the computations should be possible to perform in a purely time-recursive manner, implying no other need for a temporal memory of the past than the temporal scale-space representation itself. Furthermore, we add a complementary requirement of (iii) temporal scale covariance, meaning that under temporal scaling variations of the input, the temporal scale-space representations should also constitute mere temporal rescalings of the temporal scale-space representation computed from the original temporal signal before the temporal rescaling operation, complemented by a shift along the temporal scale axis

## Time-causal and time-recursive scale-space model for temporal signals

The problem that we consider is that we are given a temporal signal *f*(*t*) and want to define a set of successively smoothed temporal scale-space representations $L(t;\; \tau )$ for different values of a temporal scale parameter $\tau \ge 0$, as schematically illustrated in Fig. 1. We will throughout this treatment assume linearity and translational shift covariance, implying that the transformation from the original signal $f :{\mathbb {R}}\rightarrow {\mathbb {R}}$ to the temporal scale-space representation $L :{\mathbb {R}}\times {\mathbb {R}}_+ \rightarrow {\mathbb {R}}$ is given by convolution with some one-parameter family of scale-dependent convolution kernels $h :{\mathbb {R}}\times {\mathbb {R}}_+ \rightarrow {\mathbb {R}}$

1 $$\begin{aligned} L(t;\; \tau ) = (h(\cdot ;\; \tau ) * f(\cdot ))(t;\; \tau ) = \int \limits _{\xi \in {\mathbb {R}}} h(\xi ;\; \tau ) \, f(t - \xi ) \, \textrm{d}\xi . \end{aligned}$$

A crucial condition on this family of temporal scale-space representations is that the temporal scale-space representation $L(t;\; \tau _2)$ at any coarser temporal scale $t_2$ should correspond to a simplification of the temporal scale-space representation $L(t;\; \tau _1)$ at any finer temporal scale $t_1$.

Following (Lindeberg 1990), we shall measure this simplification property in terms of the number of local extrema in the signal at any temporal scale, and define a *scale-space kernel* as a kernel that obeys the property that the number of local extrema in the signal after convolution is guaranteed to not exceed the number of local extrema prior to the convolution operation, with the important qualifier that this property should hold *for any input signal*. Equivalently, this property can also be expressed by measuring the number of zero-crossings before and after the convolution operation. A scale-space kernel $h(t;\; \tau )$ is referred to as a *temporal scale-space kernel* (Lindeberg and Fagerström 1996) if it additionally satisfies $h(t;\; \tau ) = 0$ for $t < 0$, meaning that it does not require access to the future relative to any time moment.

To make the scale simplification property from finer to coarser temporal scales hold, we will assume that the family of temporal smoothing kernels $h(u;\ \tau )$ should obey the following cascade smoothing property^4^

2 $$\begin{aligned} h(\cdot ;\; \tau _2) = (\varDelta h)(\cdot ;\; \tau _1 \mapsto \tau _2) * h(\cdot ;\; \tau _1) \end{aligned}$$

for any pair of temporal scales $(\tau _1, \tau _2)$ with $\tau _2 > \tau _1$ and for some family of transformation kernels $(\varDelta h)(t;\; \tau _1 \mapsto \tau _2)$. We can then obtain a temporal scale-space representation if and only if the transformation kernel $(\varDelta h)(t;\; \tau _1 \mapsto \tau _2)$ between adjacent temporal scale levels $t_1$ and $t_2$ is always a temporal scale-space kernel.

### Classification of scale-space kernels for continuous signals

A fundamental question with regard to smoothing of temporal signals concerns what convolution kernels satisfy the conditions of being scale-space kernels.

#### Complete classification of continuous scale-space kernels

Interestingly, the class of one-dimensional scale-space kernels can be completely classified based on classical results by Schoenberg (1930, 1946, 1947, 1948, 1950, 1953, 1988), see also the excellent monograph by Karlin (1968). Summarizing the treatment in (Lindeberg 1993b, Sect. 3.5; 2016, Sect. 3.2), a continuous smoothing kernel is a scale-space kernel if and only if it has a bilateral Laplace-Stieltjes transform of the form (Schoenberg 1950)

3 $$\begin{aligned} \int \limits _{\xi = - \infty }^{\infty } e^{-s \xi } \, h(\xi ) \, \textrm{d}\xi = C \, e^{\gamma s^2 + \delta s} \prod _{i = 1}^{\infty } \frac{e^{a_i s}}{1 + a_i s} \quad \end{aligned}$$

for $-c< \text{ Re }(s) < c$ and some $c > 0$, where $C \ne 0$, $\gamma \ge 0$, $\delta$ and $a_i$ are real and $\sum _{i=1}^{\infty } a_i^2$ is convergent.

#### Basic classes of primitive scale-space kernels over a continuous signal domain

Interpreted over the temporal domain,^5^ this result means that there, beyond trivial rescaling and translation, are two main classes of one-dimensional scale-space kernels:

- convolution with *Gaussian kernels*
  4 $$\begin{aligned} h(\xi ) = e^{-\gamma \xi ^2}, \end{aligned}$$
- convolution with *truncated exponential functions*
  5 $$\begin{aligned} h(\xi ) = \left\{ \begin{array}{lcl} e^{- |\lambda | \xi } &{} &{} \xi \ge 0, \\ 0 &{} &{} \xi < 0, \end{array} \right. \quad \quad h(\xi ) = \left\{ \begin{array}{lcl} e^{|\lambda | \xi } &{} &{} \xi \le 0, \\ 0 &{} &{} \xi > 0, \end{array} \right. \end{aligned}$$
  for some strictly positive $|\lambda |$.

Moreover, the result means that a continuous smoothing kernel is a scale-space kernel *if and only if* it can be decomposed into a cascaded convolution of these primitives.

### Time-causal temporal scale-space kernels over continuous temporal domain

Among the above primitive smoothing kernels, we recognize the Gaussian kernel, which is a good and natural temporal smoothing kernel to use when analysing pre-recorded signals in offline scenarios. When analysing temporal signals in a real-time situation, or when modelling biological processes that operate in real time, we cannot, however, use a temporal smoothing kernel that requires access to information in the future relative to any time moment.

For building a time-causal temporal scale-space representation, the truncated exponential kernels are therefore the only possible primitive time-causal temporal smoothing kernels (Lindeberg and Fagerström 1996)

6 $$\begin{aligned} h_{\text{ exp }}(t;\; \mu _k) = \left\{ \begin{array}{ll} \frac{1}{\mu _k} e^{-t/\mu _k} &{} t \ge 0, \\ 0 &{} t < 0, \end{array} \right. \end{aligned}$$

where we will throughout this treatment adopt the convention of normalizing these kernels to unit $L_1$-norm. The Laplace transform of such a kernel is given by

7 $$\begin{aligned} H_{\text{ exp }}(q;\; \mu _k) = \int \limits _{t = - \infty }^{\infty } h_{\text{ exp }}(t;\; \mu _k) \, e^{-qt} \, \textrm{d}t = \frac{1}{1 + \mu _k q}. \end{aligned}$$

Coupling *K* such kernels in cascade leads to a composed kernel

8 $$\begin{aligned} h_{\text{ composed }}(\cdot ;\; \mu ) = *_{k=1}^{K} h_{\text{ exp }}(\cdot ;\; \mu _k) \end{aligned}$$

having a Laplace transform of the form

9 $$\begin{aligned} H_{\text{ composed }}(q;\; \mu )&= \int \limits _{t = - \infty }^{\infty } *_{k=1}^{K} h_{\text{ exp }}(\cdot ;\; \mu _k)(t) \, e^{-qt} \, \textrm{d}t \nonumber \\&= \prod _{k=1}^{K} \frac{1}{1 + \mu _k q}. \end{aligned}$$

The temporal mean and variance of the composed kernel is

10 $$\begin{aligned} m_K = \sum _{k=1}^{K} \mu _k, \quad \quad \tau _K = \sum _{k=1}^{K} \mu _k^2. \end{aligned}$$

The temporal mean $m_K$ is a coarse measure of the temporal delay of the time-causal temporal scale-space kernel, and the temporal variance $\tau _K$ is a measure of the temporal duration, also referred to as the temporal scale.

In terms of physical models, repeated convolution with this class of temporal scale-space kernels corresponds to coupling a series of *first-order integrators* with time constants $\mu _k$ in cascade

11 $$\begin{aligned} \partial _t L(t;\; \tau _k) = \frac{1}{\mu _k} \left( L(t;\; \tau _{k-1}) - L(t;\; \tau _k) \right) \end{aligned}$$

with $L(t;\; 0) = f(t)$, where the temporal scale-space representations for larger values of the scale parameter $t_k$ constitute successively temporally smoothed representations of each other. An important property of this type of temporal scale-space representation is that it is also *time-recursive*. The temporal scale-space representations $L(t;\; \tau _k)$ constitute a *sufficient temporal memory of the past* to compute the temporal scale-space representation and the next temporal moment, given a new input in the input signal *f*(*t*).

An important consequence of the above necessity result, is that this type of scale-space representation constitutes the *only* way to compute a time-causal temporal scale-space representation, given the requirement that the number of local extrema, or equivalently the number of zero-crossings, in the signal must not increase from finer to coarser temporal scales. In this respect, the temporal scale-space representations can be seen as gradual simplifications of each other from finer to coarser temporal scales.

**Fig. 2:**
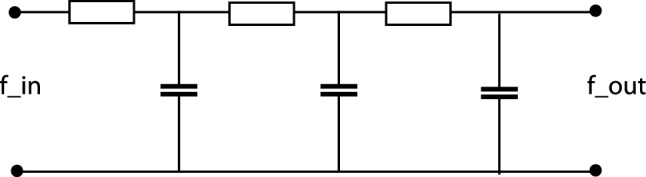
Electric wiring diagram consisting of a set of resistors and capacitors that emulate a series of first-order integrators coupled in cascade, if we regard the time-varying voltage $f_{\text{ in }}$ as representing the time varying input signal and the resulting output voltage and $f_{\text{ out }}$ as representing the time varying output signal at a coarser temporal scale. Such first-order temporal integration can be used as a straightforward computational model for temporal processing in biological neurons; see also Koch (1999, Chapters 11–12) regarding physical modelling of the information transfer in the dendrites of neurons

Figure 2 shows an illustration of this model in terms of an electric wiring diagram for transforming an input signal $f_{\text{ in }}$ to an output signal $f_{\text{ out }}$ using a set of first-order integrators coupled in cascade.

### Logarithmic distribution of the temporal scale levels

When implementing this temporal scale-space concept in practice, a set of intermediate temporal scale levels $\tau _k$ has to be distributed between some minimum and maximum temporal scale levels $\tau _{\text{ min }} = \tau _1$ and $\tau _{\text{ max }} = \tau _K$. Then, it is natural to choose these temporal scale levels according to a geometric series, corresponding to a uniform distribution in units of *effective temporal scale* $\tau _{{\text{ eff }}} = \log \tau$ (Lindeberg 1993a).

If we have a free choice of what minimum temporal scale level $\tau _{\text{ min }}$ to use, a natural way of parameterizing these temporal scale levels is by using a distribution parameter $c > 1$ such that

12 $$\begin{aligned} \tau _k = c^{2(k-K)} \tau _{\text{ max }} \quad \quad (1 \le k \le K), \end{aligned}$$

which by Eq. (10) implies that the time constants of the individual first-order integrators should be given by (Lindeberg 2016, Eqs. (19)–(20))

13 $$\begin{aligned} \mu _1&= c^{1-K} \sqrt{\tau _{\text{ max }}} \end{aligned}$$

14 $$\begin{aligned} \mu _k&= \sqrt{\tau _k - \tau _{k-1}} = c^{k-K-1} \sqrt{c^2-1} \sqrt{\tau _{\text{ max }}} \quad (2 \le k \le K). \end{aligned}$$

If the temporal signal is on the other hand given at some minimum temporal scale $\tau _{\text{ min }}$, corresponding to an *a priori* given inner temporal scale of the measurement device, we can instead determine

15 $$\begin{aligned} c = \left( \frac{\tau _{\text{ max }}}{\tau _{\text{ min }}} \right) ^{\frac{1}{2(K-1)}} \end{aligned}$$

in (12) such that $\tau _1 = \tau _{\text{ min }}$ and add $K - 1$ temporal scales with $\mu _k$ according to (14).

Temporal smoothing kernels of this form, combined with temporal differentiation for different orders of differentiation, to obtain ripples of opposite contrast in the resulting temporal receptive fields, have been used for modelling the temporal part of the processing in models for spatio-temporal receptive fields (Lindeberg and Fagerström 1996; Lindeberg 2015, 2016, 2021b) and spectro-temporal receptive fields (Lindeberg and Friberg 2015a, b).

### Logarithmic memory of the past

When using a logarithmic distribution of the temporal scale levels according to either of these methods, the different levels in the temporal scale-space representation at increasing temporal scales will serve as a logarithmic memory of the past, with qualitative similarity to the mapping of the past onto a logarithmic time axis in the scale-time model by Koenderink (1988). Such a logarithmic memory of the past can also be extended to later stages in a visual, auditory or other form of neural hierarchy.

An alternative type of temporal memory structure can be obtained if the different truncated exponential kernels are applied, not in a cascade as above, but instead in parallel with a single temporal time constant for each temporal memory channel,

16 $$\begin{aligned} h_{\text{ composed }}(\cdot ;\; \tau _k) = h_{\text{ exp }}(\cdot ;\; \mu _k) \end{aligned}$$

for $\mu _k = \sqrt{\tau _k}$, again with a logarithmic distribution of the temporal scale levels $\tau _k$. Such a model for temporal memory has been studied by Howard and his co-workers (Howard 2021; Bright et al. 2020). Then, each temporal memory channel is also a simplification of the input signal *f*(*t*), and a record of the past with a given temporal delay and temporal duration. Inversion from the temporal memory channels to the input signal is also more straightforward, from the conceptual similarity to a real-valued Laplace transform (Howard et al. 2018; Howard and Hasselmo 2020). The different temporal memory channels are, however, not guaranteed to constitute formal simplifications of each other, as they are for the cascade model.

The theoretical framework for time-causal and time-recursive temporal scale-space representations presented earlier in (Lindeberg and Fagerström 1996; Lindeberg 2016) and here can be seen as providing a theoretical foundation for such time-recursive temporal memory models.

### Uniform distribution of the temporal scale levels

An alternative approach to distributing the temporal scale levels is to use a uniform distribution of the intermediate temporal scales

17 $$\begin{aligned} \tau _k = \frac{k}{K} \, \tau _{\text{ max }}, \end{aligned}$$

implying that the time constants in the individual smoothing steps are given by

18 $$\begin{aligned} \mu _k = \mu = \sqrt{\frac{\tau _{\text{ max }}}{K}}. \end{aligned}$$

Then, a compact expression can be easily obtained for the composed convolution kernel corresponding to a cascade of *K* such kernels

19 $$\begin{aligned} h_{\text{ composed }}(t;\; \mu , K) = \frac{t^{K-1} \, e^{-t/\mu }}{\mu ^K \, \varGamma (K)}. \end{aligned}$$

**Fig. 3:**
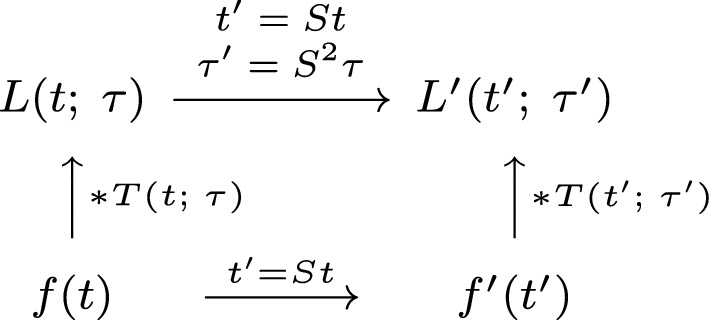
Commutative diagram for temporal receptive field responses under *temporal scaling transformations* of the temporal domain. Such transformations describe the effect of events occurring slower or faster in the world. (The commutative diagram should be read from the lower left corner to the upper right corner, and means that irrespective of whether the image is first convolved with a temporal smoothing kernel and then subject to temporal scaling transformation, or whether the temporal signal is first subject to a temporal scaling transformation and then convolved with a temporal smoothing kernel, we should get the same result provided that the temporal scale parameters $\tau$ and $\tau '$ are properly matched to the relative temporal scaling factor *S* between the two temporal patterns)

**Fig. 4:**
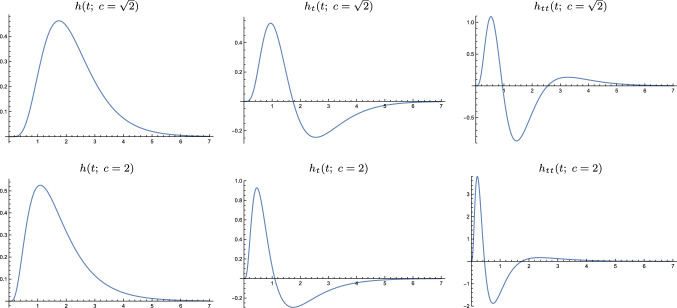
Approximations of the time-causal limit kernel for $\tau = 1$ using $K = 7$ *truncated exponential kernels* in cascade and their first- and second-order derivatives. (top row) Logarithmic distribution of the scale levels for $c = \sqrt{2}$. (bottom row) Logarithmic distribution for $c = 2$. (Horizontal axes: time. Vertical axes: function values)

**Fig. 5:**
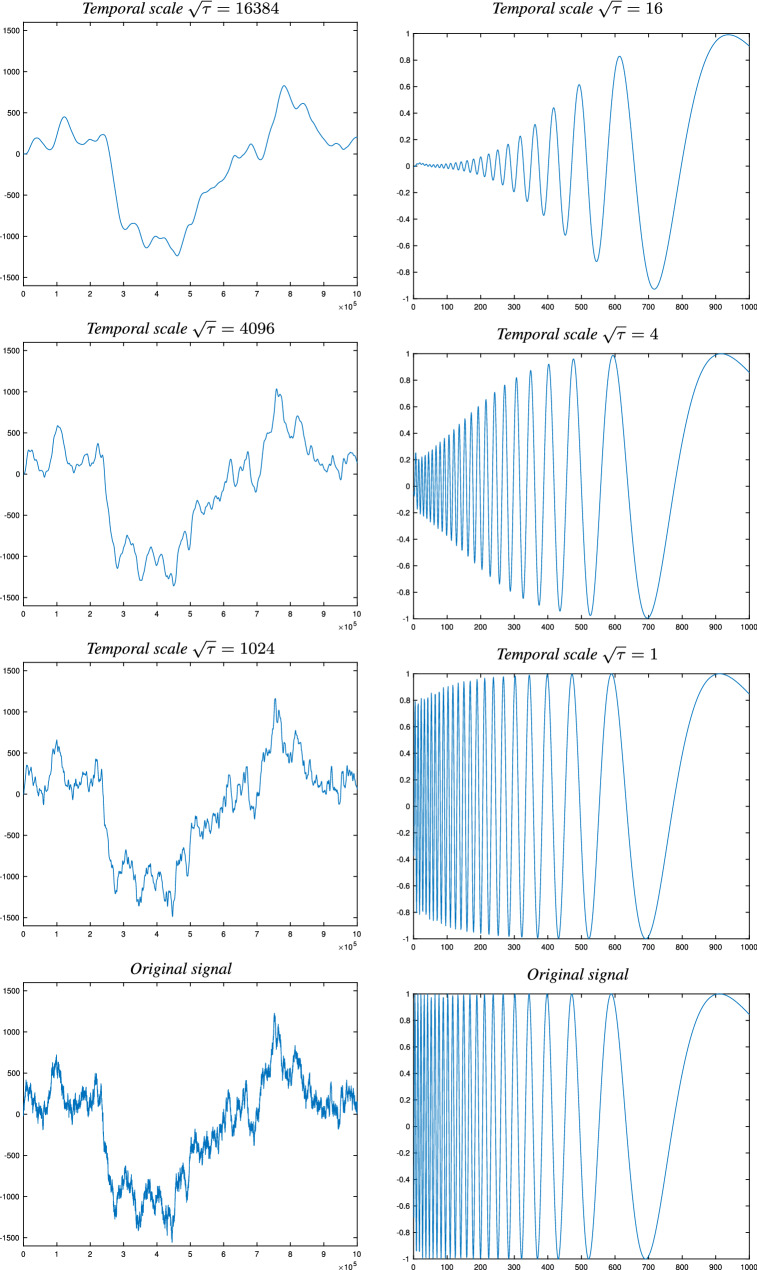
Illustration of temporal smoothing of two signals: (left) a Brownian noise signal generated from a simulated Wiener process and (right) a synthetic sine wave signal $f(t) = \sin (\exp ((b-t)/a))$ for $a = 200$ $b = 1000$ with temporally varying frequency so that the wavelength increases with time *t*, computed using a discrete approximation of the time-causal limit kernel for $c = 2$ in terms of a set of recursive filters coupled in cascade. Observe how fine-scale structures corresponding to higher frequencies are successively suppressed when going from finer to coarser temporal scales, and also that the temporal scale-space representations at coarser temporal scales are associated with longer temporal delays, in this figure seen as different offsets in the positions of the peaks in the temporal signal at different temporal scales. (Horizontal axes: time. Vertical axes: signal values)

Such kernels have also been used in memory models (Goldman 2009). The temporal Poisson model studied in more detail in (Lindeberg 1997a) can be seen as the limit case of such a uniform distribution of the temporal scale levels in the time-discrete case, when the difference between adjacent temporal scales tends to zero, a limit case that, however, only exists for discrete temporal signals (Lindeberg and Fagerström 1996), and which also serves as a multi-scale temporal memory of the past (see the illustrations of how the temporal scale-space representation evolves over time and temporal scales in the time-scale diagrams in Figs. 3–5 in (Lindeberg 1997a), which demonstrate the temporal memory properties of such a temporal scale-space representation — specifically observe the property that an event that occurs at a certain temporal moment first appears in the temporal scale-space representation at the finest temporal scale, and then moves to gradually coarser temporal scales as time passes by, and is thus also after some short times gradually forgotten at the finer temporal scales, being taken over temporal structures that appear after the initial temporal event).

For constructing temporal memory processes that are to operate over wide ranges of temporal scales, such models based on a uniform sampling of the temporal scale levels do, however, require a larger number of primitive temporal integrators, and thus more hardware or wetware, compared to a temporal memory model based on a logarithmic distribution of the temporal scale levels.

Combined with temporal differentiation of the smoothing kernel, such temporal kernels have been used for modelling the temporal response properties of neurons in the visual system (den Brinker and Roufs 1992) and for computing spatio-temporal image features in computer vision (Rivero-Moreno and Bres 2004; van der Berg et al. 2014).

For a given value of the temporal scale (the temporal variance) of such time-causal kernels, the temporal delay for a temporal kernel based on a uniform distribution of the temporal scale levels will, however, also be longer than for a temporal kernel constructed from a logarithmic distribution of the intermediate temporal scale levels. Thus, for formulating computational algorithms for expressing time-critical decision processes in computer vision or machine listening, as well as for modelling time-critical decision processes in biological perception or cognition, we argue that a logarithmic distribution of the temporal scale levels should be a much better choice.

For these reasons, we will henceforth in this treatment focus solely on models based on a logarithmic distribution of the temporal scale levels.

## Time-causal temporal scale-space representations that also obey temporal scale covariance

Beyond the task of representing temporal signals at multiple temporal scales, a main requirement on a temporal scale-space representation should also be the notion of *temporal scale covariance*,^6^ so as to be able to consistently handle temporal phenomena and events that occur faster or slower in the world. Temporal scale covariance means that if a signal *f*(*t*) is subject to a temporal scaling transformation

20 $$\begin{aligned} f'(t') = f(t) \quad \quad \text{ for } \quad \quad t' = S t \end{aligned}$$

and then processed, here with a temporal convolution kernel $T(t';\; \tau ')$ that depends on a temporal scale parameter $\tau '$,

21 $$\begin{aligned} L'(t';\; \tau ') = (T(\cdot ;\; \tau ') * f'(\cdot ))(t';\; \tau '), \end{aligned}$$

the result should be essentially similar to the result of applying the same type of processing to the original signal

22 $$\begin{aligned} L(t;\; \tau ) = (T(\cdot ;\; \tau ) * f(\cdot ))(t;\; \tau ) \end{aligned}$$

and then rescaling the processed original signal

23 $$\begin{aligned} L'(t';\; \tau ') = L(t;\; \tau ) \end{aligned}$$

(for other types of processes possibly also complemented with some minor modification, such as a correction of the magnitude of the response). For the task of temporal filtering in a temporal scale-space representation, this implies that the temporal scale-space kernel should commute with temporal scaling transformations, as illustrated in the commutative diagram in Fig. 3.

This algebraic closedness property under temporal scaling transformations will imply that similar temporal phenomena that occur faster or slower in the world will be treated in a conceptually similar manner. Under variations caused by scaling transformations in the input, the output of applying scale-covariant processing to such temporally rescaled data will be mere temporal rescalings of each other, thus without bias to any particular scales, which would otherwise be a severe shortcoming, if the computational model is not well-behaved under temporal scaling transformations.

In this section, we will describe a theory for how to obtain time-causal temporal scale-space representations that also obey such temporal scale covariance, which in turn makes it possible to construct provably scale-invariant temporal representations at higher levels in a temporal processing hierarchy. The way that we will reach this goal is by constructing a limit kernel that is the convolution of an infinite number of truncated exponential kernels in cascade, with specially chosen time constants that correspond to a geometric distribution of the intermediate temporal scale levels.

Unfortunately, there is no known simple compact explicit expression for this limit kernel in the temporal domain, implying that some of the closed-form calculations using the limit kernel may be interpreted as somewhat technical at the first encounter with this function. Once these algebraic transformation properties have been established for the limit kernel, however, this function can be handled and used in a similar way as other standard functions in mathematics.

For practical implementations, the limit kernel can furthermore for the purpose of computing the representation at a single temporal scale often be very well approximated by a moderate finite number of truncated exponential kernels coupled in cascade, usually between 4 and 8 in our implementations of this concept, because of its rapid convergence properties for suitable values of its internal distribution parameter. In turn, for the purpose of computing another temporal scale-space representation at the next coarser temporal scale, applying a *single* truncated exponential kernel to the nearest finer temporal scale is sufficient.

In this section, we will first define the limit kernel and derive its transformation properties. Then, we will turn to relating and comparing the limit kernel to two other models used for expressing temporal variations over time.

### The time-causal limit kernel

Consider the Fourier transform of the composed convolution kernel that we obtain by coupling *K* truncated exponential kernels in cascade with a logarithmic distribution of the temporal scale levels and thus time constants according to (13) and (14) for some $c > 1$:

24 $$\begin{aligned}{} & {} \hat{h}_{\text{ composed }}(\omega ;\; \tau , c, K) = \nonumber \\{} & {} \quad \frac{1}{1 + i \, c^{1-K} \sqrt{\tau } \, \omega } \prod _{k=2}^{K} \frac{1}{1 + i \, c^{k-K-1} \sqrt{c^2-1} \sqrt{\tau } \, \omega }. \end{aligned}$$

By formally letting the number of primitive smoothing steps *K* tend to infinity and renumbering the indices by a shift in terms of one unit, we obtain a limit object of the form (Lindeberg 2016, Eq. 38)

25 $$\begin{aligned} \hat{\varPsi }(\omega ;\; \tau , c)&= \lim _{K \rightarrow \infty } \hat{h}_{\text{ composed }}(\omega ;\; \tau , c, K) \nonumber \\&= \prod _{k=1}^{\infty } \frac{1}{1 + i \, c^{-k} \sqrt{c^2-1} \sqrt{\tau } \, \omega }. \end{aligned}$$

By treating this limit kernel as an object by itself, which will be well-defined because of the rapid convergence by the summation of variances according to a geometric series, interesting relations can be expressed between the temporal scale-space representations

26 $$\begin{aligned} L(t;\; \tau , c) = \int \limits _{u = 0}^{\infty } \varPsi (u;\; \tau , c) \, f(t-u) \, \textrm{d}u \end{aligned}$$

obtained by convolution with this limit kernel.

#### Self-similar recurrence relation for the time-causal limit kernel over temporal scales

Using the limit kernel, an infinite number of discrete temporal scale levels is implicitly defined given the specific choice of one temporal scale $\tau = \tau _0$:

27 $$\begin{aligned} \dots \frac{\tau _0}{c^6}, \frac{\tau _0}{c^4}, \frac{\tau _0}{c^2}, \tau _0, c^2 \tau _0, c^4 \tau _0, c^6 \tau _0, \dots \end{aligned}$$

Directly from the definition of the limit kernel, we obtain the following recurrence relation between adjacent temporal scales:

28 $$\begin{aligned} \varPsi (\cdot ;\; \tau , c) = h_{\text{ exp }}(\cdot ;\; \tfrac{\sqrt{c^2-1}}{c} \sqrt{\tau }) * \varPsi \left( \cdot ;\; \tfrac{\tau }{c^2}, c\right) \end{aligned}$$

and in terms of the Fourier transform:

29 $$\begin{aligned} \hat{\varPsi }(\omega ;\; \tau , c) = \frac{1}{1 + i \, \tfrac{\sqrt{c^2-1}}{c} \sqrt{\tau } \, \omega } \, \hat{\varPsi }\left( \omega ;\; \tfrac{\tau }{c^2}, c\right) . \end{aligned}$$

#### Behaviour under temporal rescaling transformations

From the Fourier transform of the limit kernel (25), we can observe that for any temporal scaling factor *S* it holds that

30 $$\begin{aligned} \hat{\varPsi }(\tfrac{\omega }{S};\; S^2 \tau , c) = \hat{\varPsi }(\omega ;\; \tau , c). \end{aligned}$$

Thus, the limit kernel transforms as follows under a scaling transformation of the temporal domain:

31 $$\begin{aligned} S \, \varPsi (S \, t;\; S^2 \tau , c) = \varPsi (t;\; \tau , c). \end{aligned}$$

If we, for a given choice of distribution parameter *c*, rescale the input signal *f* by a temporal scaling factor $S = 1/c$ such that $t' = t/c$, it then follows that the scale-space representation of $f'$ at temporal scale $\tau ' = \tau /c^2$

32 $$\begin{aligned} L'\left( t';\; \tfrac{\tau }{c^2}, c\right) = \left( \varPsi \left( \cdot ;\; \tfrac{\tau }{c^2}, c\right) * f'\left( \cdot \right) \right) \left( t';\; \tfrac{\tau }{c^2}, c\right) \end{aligned}$$

will be equal to the temporal scale-space representation of the original signal *f* at scale $\tau$ (Lindeberg 2016, Eq. 46)

33 $$\begin{aligned} L'(t';\; \tau ', c) = L(t;\; \tau , c). \end{aligned}$$

Hence, under a rescaling of the original signal by a temporal scaling factor *c*, a rescaled copy of the temporal scale-space representation of the original signal can be found at the next lower discrete temporal scale, relative to the temporal scale-space representation of the original signal.

#### Provable temporal scale covariance

Applied recursively, the above result implies that the temporal scale-space representation obtained by convolution with the limit kernel *obeys a closedness property over all temporal scaling transformations* $t' = c^j t$ *with temporal rescaling factors* $S = c^{j}$ ($j \in {\mathbb {Z}}$) *that are integer powers of the distribution parameter* *c* (Lindeberg 2016, Eq. 47),

34 $$\begin{aligned} L'(t';\; \tau ', c) = L(t;\; \tau , c) \quad \text{ for }\quad t' = c^j t \quad \text{ and } \quad \tau ' = c^{2j} \tau , \end{aligned}$$

thus allowing for perfect scale covariance over the restricted subset of scaling factors $S = c^j$ that precisely matches the specific set of discrete temporal scale levels that is defined by a specific choice of the distribution parameter *c*. Based on this desirable and highly useful property, it is natural to refer to the limit kernel as *the scale-covariant time-causal limit kernel* (Lindeberg 2016, Sect. 5).

**Fig. 6:**
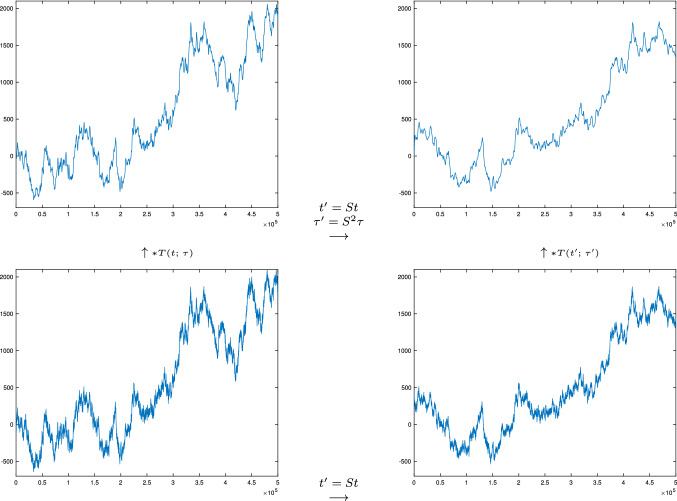
Illustration of the temporal scale covariance property of the temporal scale-space representation defined from convolutions with the time-causal limit kernel. In the bottom row, the signal in the right column is a rescaling of the signal in the left column by a temporal scaling factor $S = 2$ (with the temporal rescaling performed relative to the center of the temporal interval). In the top row, the temporal scale-space representations at the matching temporal scale levels $\sqrt{\tau } = 128$ and $\sqrt{\tau '} = 256$ have for distribution parameter $c = 2$ been computed from the corresponding input signals in the bottom row. Due to the temporal scale-covariance property, these temporal scale-space representations are in the ideal continuous case related by a temporal scaling transformation with the same temporal scaling factor $S = 2$ as between the input signals. If one for experimental purposes compares a corresponding temporal rescaling of the output from the discrete implementation in terms of recursive filters (described in more detail in Sect. 4), one can see that the corresponding graphs are practically indistinguishable (see Fig. 7). In this way, this experiment verifies and visualizes the theoretical properties reflected in the commutative diagram in Fig. 3. (Horizontal axes: time. Vertical axes: signal values)

**Fig. 7:**
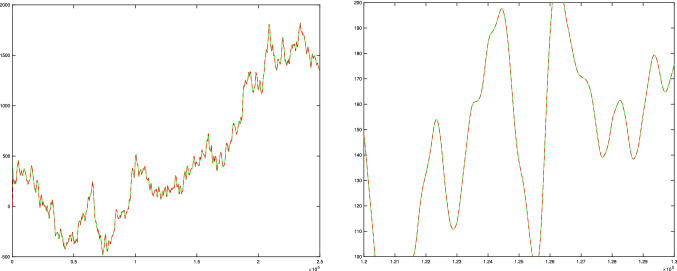
Comparison between the two different ways of computing the representation in the upper right corner in Fig. 6 from the corresponding representation in the lower left corner, using either the clockwise direction (marked in green) or the counterclockwise direction (marked in red). When generating this illustration, we have first essentially performed a rescaling of the scale-space representation of the signal in the left column and marked the result as solid green curve, and then overlayed the scale-space representation of the signal in the right column with a dashed red curve. (Technically, in the discrete implementation, we have, however, instead visualized the equivalent result of such a computation at a lower resolution, to avoid the formally ill-defined operation of interpolating the discrete signal in the left column to a higher resolution, and instead subsampled the signal in the right column, which explains the change in the labelling of the temporal axis.) (left) The result for the entire temporal interval used in the right column in Fig. 6. (right) Enlargement of a central region of the temporal interval. As can be seen from the visualization, the results computed in the clockwise or counterclockwise directions are basically indistinguishable, demonstrating the scale covariance property of the temporal scale-space representation defined by convolution with the time-causal limit kernel. (The result is best viewed by zooming in to a digital copy of the article.) (Horizontal axes: time. Vertical axes: signal values)

#### Qualitative properties

Figure 4 shows graphs of this time-causal limit kernel as well its first- and second-order temporal derivatives for a few values of the distribution parameter *c*. As can be seen from the graphs, the raw smoothing kernels have a skewed shape, where the temporal delay increases with decreasing values of the distribution parameter *c*, and with the explicit measures of the skewness $\gamma _1$ and kurtosis $\gamma _2$ of these kernels increasing as function of the distribution parameter *c* according to (Lindeberg 2016, Eqs. (130) and (131))

35 $$\begin{aligned} \gamma _1&= \frac{2 (c+1) \sqrt{c^2-1}}{\left( c^2+c+1\right) }, \end{aligned}$$

36 $$\begin{aligned} \gamma _2&= \frac{6 \left( c^2-1\right) }{c^2+1}. \end{aligned}$$

#### Experimental results

Figure 5 shows the result of smoothing two synthetic temporal signals with the time-causal limit kernel for different values of the temporal scale parameter $\tau$. As can be seen from the graphs, the signal is gradually smoothed from finer to coarser temporal scales, here clearly seen in the way that finer-scale structures are suppressed before coarser-scale structures in the left column and that higher frequencies are suppressed before lower frequencies in the right column. In addition, the temporal delay increases from finer to coarser temporal scales, here seen in terms of different temporal offsets regarding the temporal moments at which the temporal peaks occur.

When using a comparably large value of the distribution parameter *c*, as used in this figure, the temporal delay will be comparably low, which is a preferable property when needing to respond fast in a time-critical context. When using lower values of the distribution parameter, the temporal delay at a given temporal scale will be longer, which may be a preferable property if you want to use the temporal scale-space representations as temporal memory buffers, with the coarser temporal scale representations then constituting memories of what has happened further in the past.

Figure 6 gives an experimental illustration of the temporal scale covariant property of the time-causal limit kernel. Here, a synthetic signal generated from a simulated Wiener process has been rescaled by a temporal rescaling factor $S = 2$. From these two input signals, temporal scale-space representations have then been computed at the matching temporal scale levels $\sqrt{\tau } = 128$ and $\sqrt{\tau '} = 256$. Due to the temporal scale covariance property, these temporal scale-space representations are then also related by the same temporal scaling factor $S = 2$.

Figure 7 gives an illustration of the equality between the two different ways of computing the representation in the upper right corner from the signal in the lower left corner in Fig. 6, using either a clockwise orientation or a counterclockwise orientation in the corresponding commutative diagram in Fig. 3. As can be seen from the visualization, the results are essentially indistinguishable, showing that a good numerical approximation to temporal scale covariance can also be achieved in a discrete implementation (to be described further in Sect. 4).

#### Applications of the time-causal limit kernel

The time-causal limit kernel and its temporal derivatives has been used for modelling the temporal component in spatio-temporal receptive fields in the retina, the LGN and the primary visual cortex (V1) (Lindeberg 2021b), for modelling the temporal component in methods for spatio-temporal feature detection in video data (Lindeberg 2016), for expressing methods for temporal scale selection in temporal signals (Lindeberg 2017, 2018b), for modelling the temporal component of spatio-temporal smoothing in methods for spatio-temporal scale selection (Lindeberg 2018a, b) and for modelling the temporal component of smoothing in computer vision methods for video analysis (Jansson and Lindeberg 2018).

In Sect. 7.3, we do additionally propose to use the time-causal limit kernel for modelling temporal phenomena at multiple temporal scales in neural signals, and in Sect. 7.2 specifically to use this kernel for modelling the temporal variability in auditory receptive fields.

In Sect. 6.1 we outline how the time-causal limit kernel can be used for defining time-causal and time-recursive wavelet representations, and in Sect. 6.2 how the time-causal limit kernel makes it possible to define time-causal and time-recursive time-frequency representations (spectrograms) that additionally obey temporal scale covariance.

**Fig. 8:**
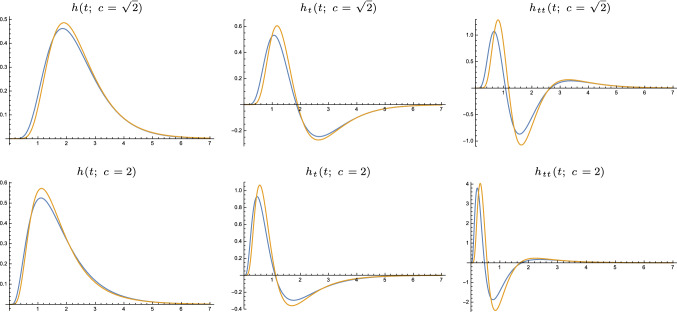
Comparison between (blue curves) the time-causal limit kernel according to (25) and approximated using the first $K = 7$ components of the infinite convolution of truncated exponential kernels in cascade with its first- and second-order temporal derivatives and (brown curves) the temporal kernels in Koenderink’s scale-time model (37) and their first- and second-order temporal derivatives. All kernels correspond to temporal scale (variance) $\tau = 1$ with the additional parameters determined such that the temporal mean values (the first-order temporal moments) become equal in the limit case when the number of temporal scale levels *K* tends to infinity (Eq. 38). (top row) Logarithmic distribution of the temporal scale levels for $c = \sqrt{2}$ (bottom row) Corresponding results for $c = 2$. (Horizontal axes: time. Vertical axes: function values)

**Fig. 9:**
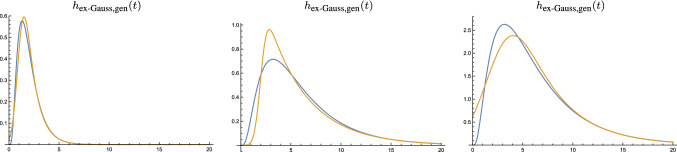
Comparison between (brown curves) the ex-Gaussian model according to (40) and (blue curves) the time-causal limit kernel according to (25) and approximated using the first $K = 7$ components of the infinite convolution of truncated exponential kernels in cascade. (left) for $\mu = 1$, $\sigma = 1/2$, $m = 1$, $a_0 = 0$ and $a_1 = 1$ corresponding to $\tau \approx 1.24$, $c \approx 1.89$, $b_0 = 0$ and $b_1 \approx 1.25$, (middle) for $\mu = 4$, $\sigma = 1/2$, $m = 2$, $a_0 = 0$ and $a_1 = 1$ corresponding to $\tau \approx 16.25$, $c \approx 2.65$, $b_0 = 0$ and $b_1 \approx 5.01$, (right) for $\mu = 4$, $\sigma = 2$, $m = 2$, $a_0 = 0$ and $a_1 = 1$ corresponding to $\tau \approx 18.94$, $c \approx 2.89$, $b_0 = 0$ and $b_1 \approx 19.37$. (Horizontal axes: time. Vertical axes: function values)

### Alternative scale-covariant temporal models

An alternative type of temporal model that one could also consider from the general classification of temporal scale-space kernels is to use a set of parallel temporal channels formed by convolution of the input signal, with a single truncated exponential function in each channel, and with a geometric distribution of the their time constants, of the form (16). As previously explained in Sect. 2.4, such temporal models have been previously used as models of temporal memory in neuroscience (Howard 2021; Bright et al. 2020).

Because of the geometric distribution of the time constants in these temporal channels, they will obey temporal scale covariance. Temporal scale covariance will also apply to different types of generalizations of such a model, *e.g.* by having the same small number of truncated exponential kernels in cascade in each temporal channel, with the time constants between the different temporal channels coupled according to a geometric distribution.

A fundamental difference between such temporal models and the temporal scale-space model based on the time-causal limit kernel, however, is that in the first class of models the temporal channels for larger values of the scale parameter are not guaranteed to constitute simplifications of the temporal channels for smaller values of the scale parameter. By the temporal smoothing kernels being scale-space kernels, each temporal channel is guaranteed to be a simplification of the input signal. When relating different temporal scale channels to each other, however, the number of local extrema in a temporal channel for a larger value of the temporal scale parameter is not guaranteed to not exceed the number of local extrema in a temporal channel for a finer smaller value of the temporal scale parameter.

Because of the scale-recursive property (28) of the time-causal limit kernel, it is on the other hand formally guaranteed that the temporal scale-space representation at the next coarser temporal scale corresponds to the result of applying temporal smoothing with a truncated exponential kernel to the temporal scale-space representation at the nearest finer temporal scale. Applied recursively, the temporal scale-space representation at any coarser temporal scale corresponds to the result of applying a set of truncated exponential kernels in cascade to the representation at any finer temporal scale. In this way, for the temporal scale-space representation generated by convolution with the time-causal limit kernel for different values of the temporal scale parameter, every temporal scale-space representation at a given temporal scale is guaranteed to constitute a formal simplification of any other temporal scale-space representation at any finer temporal scale.

The time-causal limit kernel is special in that it both obeys temporal scale covariance and guarantees non-creation of new local extrema with increasing temporal scales with regard to convolutions over a time-causal temporal domain.

### Relation to Koenderink’s scale-time model

In his scale-time model, Koenderink (1988) proposed to perform a logarithmic mapping of the past via a temporal delay $\delta$ and then applying Gaussian smoothing with standard deviation $\sigma$ in the transformed domain. If we additionally normalize these kernels to unit $L_1$-norm, we obtain a time-causal kernel of the form (Lindeberg 2016, Eq. 151)

37 $$\begin{aligned} h_{\text{ Koe }}(t;\; \sigma , \delta ) =\frac{1}{\sqrt{2 \pi } \sigma \,\delta } e^{-\frac{\log ^2\left( \frac{t}{\delta }\right) }{2 \sigma ^2} -\frac{\sigma ^2}{2}}. \end{aligned}$$

In (Lindeberg 2016, Appendix 2) a formal mapping between this scale-time kernel and the time-causal limit kernel is derived, by requiring the first- and second-order moments of these two classes of kernels to be equal:

38 $$\begin{aligned} \left\{ \begin{array}{l} \tau = \delta ^2 \, e^{3 \sigma ^2} \left( e^{\sigma ^2}-1\right) \\ c = \frac{e^{\sigma ^2}}{2-e^{\sigma ^2}} \end{array} \right. \quad \quad \left\{ \begin{array}{l} \sigma = \sqrt{\log \left( \frac{2 c}{c+1}\right) } \\ \delta = \frac{(c+1)^2 \sqrt{\tau }}{2 \sqrt{2} \sqrt{(c-1) c^3}} \end{array} \right. \end{aligned}$$

which hold as long as $c > 1$ and $\sigma < \sqrt{\log 2} \approx 0.832$.

Figure 8 shows a comparison between the time-causal limit kernel and Koenderink’s scale-time kernels regarding the zero-order convolution kernels as well as their first- and second-order derivatives. As can be seen from the graphs, these two classes of kernels have qualitatively rather similar shapes. The time-causal limit kernel does, however, have the conceptual advantage that it can be computed in a time-recursive manner, whereas the scale-time kernel does not have any known time-recursive implementation, implying that it formally requires an infinite memory of the past (or some substantially extended temporal buffer, if the infinite temporal convolution integral is truncated at the tail).

While we do not have any compact explicit expression for the time-causal limit kernel over the temporal domain, if we approximate the time-causal limit kernel by a scale-time kernel according to the mapping (38), we obtain the following estimate for the location of the maximum point of the time-causal limit kernel:

39 $$\begin{aligned} t_{\text{ max }} \approx \frac{(c+1)^2 \, \sqrt{\tau }}{2 \sqrt{2} \sqrt{(c-1) c^3}} = \delta . \end{aligned}$$

This estimate can be expected to be an overestimate, and is a better estimate of the temporal delay of the time-causal limit kernel than the temporal mean according to (10).

### Relation to the ex-Gaussian model used by Bright et al.

In (Bright et al. 2020), a so-called ex-Gaussian model (Grushka 1972), that is the convolution of an unnormalized Gaussian function with an unnormalized truncated exponential kernel

40 $$\begin{aligned} h_{\text{ ex-Gauss,gen }}(t) = a_0 + a_1 \int \limits _{u=0}^{\infty } e^{-\frac{(t-m-u)^2}{2 \sigma ^2}} e^{-\frac{u}{\mu }} \, du, \end{aligned}$$

is used for fitting temporal response functions of neurons to an analytical temporal model. In Appendix A.1, a relation between this ex-Gaussian model and a corresponding model based on the time-causal limit kernel

41 $$\begin{aligned} h_{\text{ limit-kern,gen }}(t) = b_0 + b_1 \, \varPsi (t;\; \tau , c) \end{aligned}$$

is derived by requiring the zero-, first- and second-order temporal moments of these kernels to be equal, if the DC-offsets $a_0$ and $b_0$ are disregarded and assumed to be equal.

This leads to the following mapping between the parameters of the two models

42 $$\begin{aligned}&b_1 = M_0, \end{aligned}$$

43 $$\begin{aligned}&c = \frac{\delta ^2+V}{\delta ^2-V}, \end{aligned}$$

44 $$\begin{aligned}&\tau = V, \end{aligned}$$

where $\delta$ and *V* denote the temporal mean and the temporal variance of the ex-Gaussian model for $a_0 = 0$

45 $$\begin{aligned} \delta&= \frac{M_1}{M_0}, \end{aligned}$$

46 $$\begin{aligned} V&= \frac{M_2}{M_0} - \left( \frac{M_1}{M_0} \right) ^2, \end{aligned}$$

and $M_0$, $M_1$ and $M_2$ denote the explicit expressions for the zero-, first- and second-order moments of the ex-Gaussian model for $a_0 = 0$, according to (88), (89) and (90).

Figure 9 shows a few examples of ex-Gaussian temporal models approximated by models based on the time-causal limit kernel in this way. As can be seen from the graphs, the two classes of kernels can capture qualitative similar temporal shapes in time-causal temporal data,^7^ with the conceptual differences that: (i) the model based on the time-causal limit kernel always tends to zero at the temporal origin $t = 0$ when the DC-offset is zero, whereas the ex-Gaussian model may take nonzero values for $t = 0$, (ii) the time-causal limit kernel does not contain any internal non-causal temporal component as the time-shifted Gaussian kernel in (40) constitutes, and (iii) the time-causal limit kernel has a completely time-recursive implementation, which is essential when modelling temporal phenomena in real time as they, for example, occur in biological neurons. The model based on the time-causal limit kernel is also specifically possible to implement based on a cascade of first-order integrators in cascade, which is a natural model for the information transfer in the dendrites of neurons (Koch 1999, Chapters 11–12).

#### Extension to third-order moment-based model fitting involving also a flexible temporal offset

In Appendix A.2, an extension of the above second-order moment-based model to a third-order moment-based model is performed, which makes it possible to also determine a temporal offset $t_0$

47 $$\begin{aligned} h_{\text{ limit-kern,gen }}(t) = b_0 + b_1 \, \varPsi (t-t_0;\; \tau , c), \end{aligned}$$

and which may be relevant in situations when the temporal origin of the signal cannot be accurately determined in an experimental situation. Since the closed-form expressions for the solutions become more complex in this case (they are determined from the solutions of a fourth-order algebraic equation), we restrict ourselves to a conceptual and algorithmic description in this treatment, see Appendix A.2 for further theoretical details and experimental results.

#### Extension to model fitting for other signals or functions

The above general procedures, whereby the parameters in the model based on the time-causal limit kernel are determined from the lower-order temporal moments of the data, can also be more generally used for fitting models based on the time-causal limit kernel to other signals and functions that: (i) are defined for non-negative values of time, (ii) assume non-negative values only, (iii) have a roughly unimodal shape of first increasing and then decreasing and (iv) decay towards zero towards infinity. The approach for fitting basically implies replacing the temporal moments $M_0$, $M_1$, $M_2$ and optionally $M_3$ of the ex-Gaussian model by the temporal moments of the signal or function to be fit with a model based on the time-causal limit kernel, see Appendix A.3 for additional details.

## Computational implementation of convolutions with the time-causal limit kernel on discrete temporal data

In the theory presented so far, we have throughout assumed that the signal is continuous over time. When implementing this model on sampled temporal data, the theory must be transferred to a discrete temporal domain.

In this section, we will describe how the temporal receptive fields can be implemented in terms of corresponding discrete temporal scale-space kernels that possess scale-space properties over a discrete temporal domain, and in addition are both time-causal and fully time-recursive.

Following Lindeberg (1990) and in a corresponding way as the treatment in Sect. 2, let us define a discrete kernel as a discrete scale-space kernel if for any input signal it is guaranteed that the number of local extrema, alternatively the number of zero-crossings, cannot increase under convolution with the discrete scale-space kernel.

### Classification of scale-space kernels for discrete signals

To characterize the class of discrete scale-space kernels, we can, in a corresponding way as for the continuous case, also build upon classical results by Schoenberg (1930, 1946, 1947, 1948, 1950, 1953, 1988), and as further developed in the monograph by Karlin (1968).

Making a summary of the treatment in Lindeberg (1990, Sect. IV) (2016, Sect. 6.1), a discrete smoothing kernel is a discrete scale-space kernel if and only if it has its generating function of the sequence of filter coefficients $\varphi (z) = \sum _{n=-\infty }^{\infty } c_n z^n$ of the form (Schoenberg 1948)

48 $$\begin{aligned} \varphi (z) = c \; z^k \; e^{(q_{-1}z^{-1} + q_1z)} \prod _{i=1}^{\infty } \frac{(1+\alpha _i z)(1+\delta _i z^{-1})}{(1-\beta _i z)(1-\gamma _i z^{-1})} \end{aligned}$$

where $$c > 0$$, $$k \in {\mathbb {Z}}$$, $$q_{-1}, q_1, \alpha _i, \beta _i, \gamma _i, \delta _i \ge 0$$ and $$\sum _{i=1}^{\infty }(\alpha _i + \beta _i + \gamma _i + \delta _i) < \infty $$.

#### Basic classes of primitive scale-space kernels over a discrete signal domain

With regard to the original temporal domain,^8^ this characterization means that, besides trivial rescalings and translations, there are three basic classes of discrete smoothing transformations:

- two-point weighted average or *generalized binomial smoothing*
  49 $$\begin{aligned} \begin{aligned} f_{\text{ out }}(x)&= f_{\text{ in }}(x) + \alpha _i \, f_{\text{ in }}(x - 1) \quad (\alpha _i \ge 0),\\ f_{\text{ out }}(x)&= f_{\text{ in }}(x) + \delta _i \, f_{\text{ in }}(x + 1) \quad (\delta _i \ge 0), \end{aligned} \end{aligned}$$
- moving average or *first-order recursive filtering*
  50 $$\begin{aligned} \begin{aligned} f_{\text{ out }}(x)&= f_{\text{ in }}(x) + \beta _i \, f_{\text{ out }}(x - 1) \quad (0 \le \beta _i< 1), \\ f_{\text{ out }}(x)&= f_{\text{ in }}(x) + \gamma _i \, f_{\text{ out }}(x + 1) \quad (0 \le \gamma _i < 1), \end{aligned} \end{aligned}$$
- *infinitesimal smoothing*^9^ or diffusion as arising from the continuous semi-groups made possible by the factor $e^{(q_{-1}z^{-1} + q_1z)}$.

To transfer the continuous first-order integrators derived in Sect. 2.2 to a discrete implementation, we shall in this treatment focus on the first-order recursive filters (50), which by additional $l_1$-normalization constitute both the discrete correspondence and a numerical approximation of time-causal and time-recursive first-order temporal integration (11).

### Discrete temporal scale-space kernels based on recursive filters

Given a signal that has been sampled by some temporal frame rate *r*, the temporal scale $\sigma _t$ in the continuous model in units of seconds is first transformed to a temporal variance $\tau$ relative to a unit time sampling

51 $$\begin{aligned} \tau = r^2 \, \sigma _t^2. \end{aligned}$$

Then, a discrete set of intermediate temporal scale levels $\tau _k$ is defined by (12) or (17), with the difference between successive scale levels according to

52 $$\begin{aligned} \varDelta \tau _k = \tau _k - \tau _{k-1} \end{aligned}$$

with $$\tau _0 = 0$$.

For implementing the temporal smoothing operation between two such adjacent scale levels (with the lower level in each pair of adjacent scales referred to as $f_{\text{ in }}$ and the upper level as $f_{\text{ out }}$), we make use of a *first-order recursive filter* normalized to the form

53 $$\begin{aligned} f_{\text{ out }}(t) - f_{\text{ out }}(t-1) = \frac{1}{1 + \mu _k} \, (f_{\text{ in }}(t) - f_{\text{ out }}(t-1)) \end{aligned}$$

and having a generating function of the form

54 $$\begin{aligned} H_{\text{ geom }}(z) = \frac{1}{1 - \mu _k \, (z - 1)}, \end{aligned}$$

which is a time-causal kernel and satisfies discrete scale-space properties of guaranteeing that the number of local extrema or zero-crossings in the signal will not increase with increasing scale (Lindeberg 1990; Lindeberg and Fagerström 1996). These recursive filters are the discrete analogue of the continuous first-order integrators (11).

Each primitive recursive filter (53) has temporal mean value $m_k = \mu _k$ and temporal variance $\varDelta \tau _k = \mu _k^2 + \mu _k$, and we compute $\mu _k$ from $\varDelta \tau _k$ in (52) according to

55 $$\begin{aligned} \mu _k = \frac{\sqrt{1 + 4 \varDelta \tau _k}-1}{2}. \end{aligned}$$

By the additive property of variances under convolution, the discrete variances of the discrete temporal scale-space kernels will perfectly match those of the continuous model, whereas the temporal mean values and the temporal delays may differ somewhat. If the temporal scale $\tau _k$ is large relative to the temporal sampling distance, the discrete model should be a good approximation in this respect.

By the time-recursive formulation of this temporal scale-space concept, the computations can be performed based on a compact temporal buffer over time, which contains the temporal scale-space representations at temporal scales $\tau _k$, and with no need for storing any additional temporal buffer of what has occurred in the past, to perform the corresponding temporal smoothing operations.

For practical implementations, we often approximate the time-causal limit kernel using 4–8 layers of recursive filters coupled in cascade using either $c = \sqrt{2}$ or $c = 2$.

A summarizing algorithmic description of how to implement these temporal filtering operations in practice is given in Appendix B.

## Computation of temporal scale-space derivatives

So far, we have been concerned with the problem of how to smooth a temporal signal in such a way that the smoothing transformation is guaranteed to not increase the number of local extrema in the signal, or equivalently the number of zero-crossings. In many applications, one is, however, more interested in studying the *change* in the signal over time, as can be modelled by temporal derivatives.

For a purely time-dependent signal, the first-order temporal derivative will lead to strong responses in the signal when the temporal slope is high, corresponding to, *e.g.* onsets or offsets of a sound in auditory processing, or motion in the world, alternatively changes in the illumination, for video processing. Regarding visual processing over a purely spatial domain, first-order spatial derivatives will respond to edges in the image domain, which in turn may correspond to discontinuities in either depth, surface orientation, reflectance or illumination in the world.

For a purely time-dependent signal, the second-order derivatives may on the other hand often lead to strong responses near local maxima or minima over time, if the sign of the first-order temporal derivative changes rapidly at those points. Concerning audio processing, a second-order temporal derivative applied to a spectrogram representation may give a strong response to, *e.g.* a beep or some other brief temporal sound, provided that the temporal scale is sufficiently near the temporal duration of the sound. Applying second-order derivatives with respect to logarithmic frequencies to a spectrogram will in turn enhance spectral bands and formants, provided that the logspectral scales are appropriately selected. Regarding visual processing, a second-order temporal derivative applied to a video stream may give a strong response to a flashing light, again assuming that the temporal scale is sufficiently near the temporal duration of the flash. Assuming that the visual observer does not fixate a moving object, second-order temporal derivatives may also give strong responses to image patterns that move relative to the viewing direction. For visual processing on a purely spatial domain, second-order spatial derivative operators can be specially designed to give strong responses to blob-like or corner-like image structures, which can be detected by interest point detectors.

Beyond such pointwise or regionwise responses over time, as described above, temporal derivatives can also be interpreted and used densely, for every time moment, and, for example, be combined according to a local Taylor expansion around any temporal moment $t_0$:

56 $$\begin{aligned} L(t_0+ \varDelta t;\; \tau )&= L(t_0;\; \tau ) + \varDelta t \, L_t(t_0;\; \tau ) \nonumber \\&\quad + \frac{(\varDelta t)^2}{2} \, L_{tt}(t_0;\; \tau ) + \mathcal{O}((\varDelta t)^3), \end{aligned}$$

to characterize the local temporal structures in the temporal signal at any scale $\tau$. Such a representation involving temporal derivatives up to order *N* is referred to as a temporal *N*-jet representation.

A practical complication that, however, arises, when computing temporal derivatives at multiple scales concerns how to compare the responses between different levels of scale. Due to the temporal smoothing operation, the amplitude of the temporal derivatives can be expected to decrease monotonically with increasing amount of temporal smoothing, provided that the temporal smoothing operation is sufficiently well-designed. This does, for example, hold for temporal smoothing with the truncated exponential kernels, which arise as the only possible temporal smoothing primitives in the time-causal scale-space kernels, including the time-causal limit kernel.

In this section, we will describe a way to reduce the problem of decreasing amplitude of temporal derivatives with increasing values of the temporal scale parameter, by instead using scale-normalized temporal derivatives. The intention is that by using appropriately designed scale-normalized derivative operators, it should be possible to judge if a temporal derivative response of a certain order at a certain temporal scale should be regarded as stronger or weaker than a corresponding temporal derivative response at some other temporal scale. We will also describe how temporal scale covariance can be obtained for temporal derivative operators that are combined with the time-causal limit kernel.

**Fig. 10:**
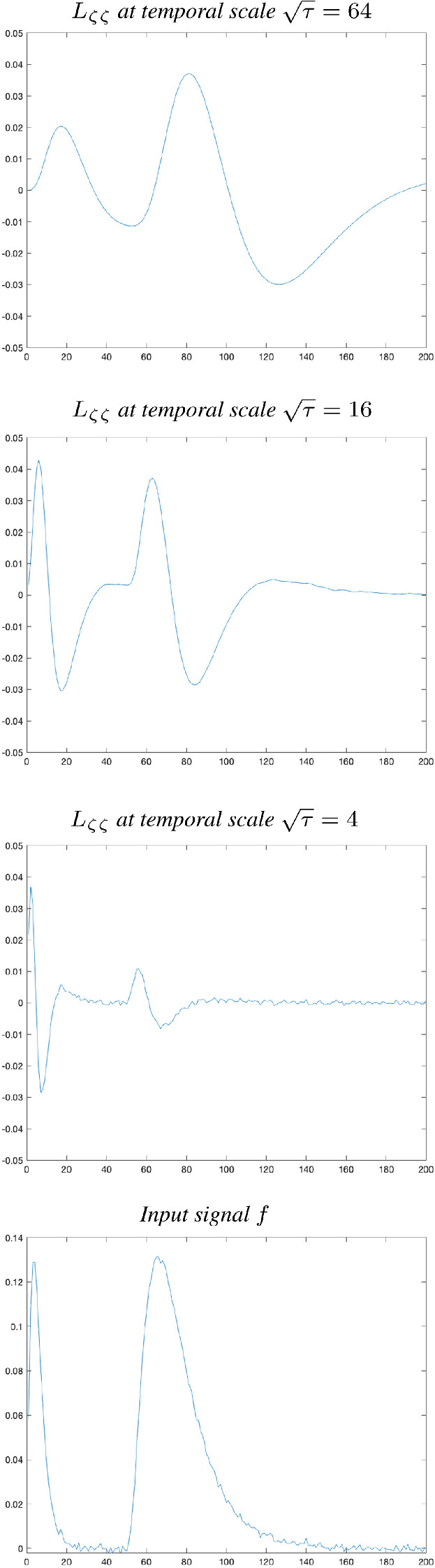
Illustration of the result of computing discrete approximations of second-order scale-normalized temporal derivatives $L_{\zeta \zeta }$ from the time-causal temporal scale-space representation *L* at different scales (using distribution parameter $c = 2$ and scale normalization power $\gamma = 1$), here for a synthetic input signal *f* consisting of two temporal peaks generated as discrete approximations to time-causal limit kernels for temporal scales $\tau = 16$ and $\tau = 256$ with a certain amount of relative temporal delay to separate the responses as well as a small amount of added white Gaussian noise. (Horizontal axes: time. Vertical axes: Signal values)

### The scale-normalized derivative concept

For the non-causal Gaussian scale-space concept defined over a purely spatial domain, and corresponding to Gaussian smoothing at all scales, it can be shown that the canonical way of defining scale-normalized derivatives at different spatial scales *s* is according to (Lindeberg 1998a, b, 2021a)

57 $$\begin{aligned} \partial _{\xi } = s^{\gamma /2} \, \partial _{x}, \end{aligned}$$

where $\gamma$ is a free parameter. Specifically, it can be shown (Lindeberg 1998a, Sect. 9.1) that this notion of $\gamma$-normalized derivatives corresponds to normalizing the *m*:th order Gaussian derivatives $g_{\xi ^m}$ over *N*-dimensional image space to constant $L_p$-norms over scale

58 $$\begin{aligned} \Vert g_{\xi ^m}(\cdot ;\; s) \Vert _p = \left( \,\, \int \limits _{t \in {\mathbb {R}}} |g_{\xi ^m}(x;\; s)|^p \, \textrm{d}t \right) ^{1/p} = G_{m,\gamma } \end{aligned}$$

with the power *p* in the $L_p$-norm depending on the scale normalization power $\gamma$, the order of differentiation *m* and the spatial dimensionality *N* of the signal according to

59 $$\begin{aligned} p = \frac{1}{1 + \frac{m}{N} \, (1 - \gamma )}, \end{aligned}$$

where the perfectly scale-invariant case $\gamma = 1$ corresponds to $L_1$-normalization for all orders *m*.

### Scale normalization for time-causal temporal derivatives

For temporal derivatives^10^ defined from the time-causal scale-space concept corresponding to convolution with truncated exponential kernels coupled in cascade, it can be shown to be meaningful to define time-causal scale-space derivatives in a corresponding manner (Lindeberg 2016, 2017):

- By *variance-based scale normalization*, we define scale-normalized temporal derivatives according to
  60 $$\begin{aligned} \partial _{\zeta ^n} = \tau ^{n \gamma /2} \, \partial _{t^n}, \end{aligned}$$
  where $\tau$ denotes the variance of the temporal smoothing kernel.
- By $L_p$*-norm-based scale normalization*, we determine a temporal scale normalization factor $\alpha _{n,\gamma }(\tau )$
  61 $$\begin{aligned} \partial _{\zeta ^n} = \alpha _{n,\gamma }(\tau ) \, \partial _{t^n} \end{aligned}$$
  such that the $L_p$-norm (with *p* determined as function of $\gamma$ according to (59)) of the corresponding composed scale-normalized temporal derivative computation kernel $\alpha _{n,\gamma }(\tau ) \, h_{t^n}$ equals the $L_p$-norm of some other reference kernel, where we may initially take the $L_p$-norm of the corresponding Gaussian derivative kernels (Lindeberg 2016, Sect. 7.3)
  62 $$\begin{aligned} \Vert \alpha _{n,\gamma }(\tau ) \, h_{t^n}(\cdot ;\; \tau ) \Vert _p&= \alpha _{n,\gamma }(\tau ) \, \Vert h_{t^n}(\cdot ;\; \tau ) \Vert _p \nonumber \\&= \Vert g_{\xi ^n}(\cdot ;\; \tau ) \Vert _p = G_{n,\gamma }. \end{aligned}$$

### Scale covariance property of scale-normalized temporal derivatives

In the special case when the temporal scale-space representation is defined by convolution with the scale-covariant time-causal limit kernel according to (26) and (25), it is shown in (Lindeberg 2016, Appendix 3) that the corresponding scale-normalized derivatives become truly scale covariant under temporal scaling transformations $t' = c^j t$ with scaling factors $S = c^j$ that are integer powers of the distribution parameter *c*

63 $$\begin{aligned} L'_{\zeta '^n}(t';\, \tau ', c)&= c^{j m (\gamma -1)} \, L_{\zeta ^n}(t;\, \tau , c) \nonumber \\&= c^{j (1 - 1/p)} \, L_{\zeta ^n}(t;\, \tau , c) \end{aligned}$$

between matching temporal scale levels $\tau ' = c^{2j} \tau$. Specifically, for $\gamma = 1$ corresponding to $p = 1$ the magnitude values of the scale-normalized temporal derivatives at matching scales become fully scale invariant

64 $$\begin{aligned} L'_{\zeta '^n}(t';\, \tau ', c) = L_{\zeta ^n}(t;\, \tau , c), \end{aligned}$$

allowing for well-defined comparisons between the magnitude values of different types of temporal structures in a signal at different temporal scales.

### A canonical class of time-causal, time-recursive and scale-covariant temporal basis functions

The above scale covariance property implies that the scale-normalized temporal derivatives of the time-causal limit kernel constitute a canonical class of temporal basis functions over a time-causal temporal domain.

These kernels have been used as temporal basis functions for spatio-temporal receptive fields (Lindeberg 2016, 2021b; Jansson and Lindeberg 2018) and for expressing methods for temporal scale selection (Lindeberg 2017, 2018b) and spatio-temporal scale selection (Lindeberg 2018a, b) that detect and compare temporal structures at different temporal scales in a completely scale-invariant manner.

In this treatment, we additionally propose to use this family of temporal basis functions to model the temporal variability of neurons over multiple scales (Sect. 7.3) and specifically the temporal variability in computational models of auditory receptive fields (Sect. 7.2).

### Discrete approximations of scale-normalized temporal scale-space derivatives

For the discrete temporal scale-space concept over discrete time described in Sect. 4.2, discrete approximations of temporal derivatives are obtained by applying temporal difference operators

65 $$\begin{aligned} \delta _t = (-1, +1), \quad \quad \delta _{tt} = (1, -2, 1) \end{aligned}$$

to the discrete temporal scale-space representation at any temporal scale, which in turn is constructed from a cascade of first-order recursive filters of the form (53), with the time constants $\mu _k$ given by (55) from the differences in temporal scale levels $\varDelta \tau _k = \tau _k - \tau _{k-1}$ with $\tau _k$ according to (12).

Scale normalization factors for discrete $l_p$-normalization are then defined in an analogous way as for continuous signals, (60) or (61), with the only difference that the continuous $L_p$-norm is replaced by a discrete $l_p$-norm.

#### Experimental results

Figure 10 shows an illustration of computing discrete approximations of second-order scale-normalized temporal derivatives in this way,^11^ for a synthetic input signal consisting of two temporal peaks generated from discrete approximations of the time-causal limit kernel for $\tau = 16$ and $\tau = 256$, respectively, and with some amount of relative temporal delay to separate the responses as well as a small amount of added white Gaussian noise.

Observe how the dominant responses to the finer-scale structures in the input signal are obtained at finer levels of scale in the temporal scale-space representation, whereas the dominant responses to the coarser-scale structures in the input signal are obtained at coarser levels of scale in the temporal scale-space.

Do also observe how the responses at coarser temporal scales are associated with longer temporal delays, manifesting themselves as temporal peaks corresponding to the underlying signal structures appearing at later time moments at coarser levels of scale.

Do furthermore note that the range of values on the vertical axis in these graphs is the same for all the scale values, demonstrating the ability to make relative comparisons between the magnitudes of the derivative responses at different scales, due to the notion of scale normalization of the temporal derivatives, here with regard to the $l_1$-norm.

## Relations to wavelet analysis and time-frequency analysis

For analyzing temporal signals at multiple temporal scales, wavelet analysis (Grossmann and Morlet 1984; Mallat 1989, 1999; Heil and Walnut 1989; Meyer 1992; Daubechies 1992; Chui 1992; Rioul and Duhamel 1992; Graps 1995; Debnath and Shah 2002) and time-frequency analysis (Gabor 1946; Cohen 1995; Feichtinger and Strohmer 1998; Qian and Chen 1999; Gröchenig 2001; Flandrin 2018) constitute two other main classes of conceptual tools. In this treatment, we do, however, not follow those notions as prototype models, instead adhering to the scale-space paradigm because of its special properties. Nevertheless, the presented temporal scale-space theory can be related to wavelet analysis and time-frequency analysis in the following ways:

### Relations to wavelet analysis

By construction, the temporal derivatives of the time-causal limit kernel $\varPsi (t;\; \tau , c)$ defined from (25) have integral equal to zero

66 $$\begin{aligned} \int \limits _{t = -\infty }^{\infty } (\partial _{t^n} \varPsi )(t;\; \tau , c) \, \textrm{d}t = 0. \end{aligned}$$

In this respect, the temporal derivatives of the time-causal kernel, complemented by normalization with respect to a suitably chosen norm, can serve^12^ as a mother wavelet over a continuous time-causal temporal domain,

67 $$\begin{aligned} W(t;\; \tau , c) = \frac{(\partial _{t^n} \varPsi )(t;\; \tau , c)}{\Vert (\partial _{t^n} \varPsi )(t;\; \tau , c) \Vert }, \end{aligned}$$

in a similar way as Gaussian derivative kernels of a certain order

68 $$\begin{aligned}{} & {} W(t;\; \sigma ) = \frac{(\partial _{t^n} g)(t;\; \sigma )}{\Vert (\partial _{t^n} g)(t;\; \sigma ) \Vert } \quad \text{ with }\quad \nonumber \\{} & {} \quad g(t;\; \sigma ) = \frac{1}{\sqrt{2\pi } \sigma } e^{-t^2/2\sigma ^2}, \end{aligned}$$

such as the Mexican hat wavelet (Marr 1976, 1982), also known as a Ricker wavelet (Ricker 1944; Hosken 1988), and corresponding to the second-order derivative of the Gaussian, can serve as a mother wavelet over a continuous non-causal temporal domain.

In wavelet analysis, one usually normalizes both the mother wavelet and the child wavelets to unit $L_2$-norm, leading to translated and rescaled child wavelets of the form

69 $$\begin{aligned} \psi _{a,b}(t) = \frac{1}{\sqrt{a}} \, W\left( \frac{t-b}{a}\right) . \end{aligned}$$

In scale-space theory, the most common way of normalizing the Gaussian derivative kernels as well as temporal derivatives of the time-causal limit kernel is to constant $L_1$-norm over scales (and corresponding to scale-normalized derivatives for $\gamma = 1$ according to Sect. 5.1), although other scale normalizations, including $L_2$-normalization, are also possible, as further described in Sect. 5.1. Such $L_1$-normalization then leads to translated and rescaled child wavelets of the form

70 $$\begin{aligned} \psi _{a,b}(t) = \frac{1}{a} \, W\left( \frac{t-b}{a}\right) . \end{aligned}$$

In the following, we will describe how the corresponding wavelet representations obtained my mapping a signal *f* onto the child wavelets can be computed if the mother wavelet is chosen as a temporal derivative of the time-causal limit kernel.

#### Handling the transformation properties of the child wavelets within the algebra of the time-causal temporal scale-space representation

By using the transformation properties of scale-normalized derivatives of the time-causal scale-space representation of the time-causal limit kernel (63), it follows that under a scaling transformation of time $t' = c^j t$ for some integer *j* with *c* being the distribution parameter of the time-causal limit kernel, and with a corresponding transformation of the temporal scale parameter $\tau ' = c^{2j} \tau$, similar transformation properties hold for the scale-normalized temporal derivatives of the time-causal limit kernel (let the input signal be the continuous delta function $f(t) = \delta (t)$ in (63))

71 $$\begin{aligned} \varPsi '_{\zeta '^n}(t';\, \tau ', c)&= c^{j m (\gamma -1)} \, \varPsi _{\zeta ^n}(t;\, \tau , c) \nonumber \\&= c^{j (1 - 1/p)} \, \varPsi _{\zeta ^n}(t;\, \tau , c), \end{aligned}$$

where $\gamma$ is the power in the temporal scale-normalized derivative concept and *p* is the power in the corresponding $L_p$-norm that is kept constant over scale by the scale-normalized derivatives.

This implies that if we choose the mother wavelet as a temporal derivative of the time-causal limit kernel according to (67), then the temporal scaling and translation operations of the child wavelets in (69) and (70) can be expressed fully within the algebra of the time-causal scale-space representation, provided that the temporal scaling factors *a* are chosen as integer powers of the distribution parameter *c* in the time-causal limit kernel according to $a = c^j$. This does in turn imply that the result of expanding a temporal test signal onto the child wavelets can be *directly extracted* as the corresponding temporal derivatives of the time-causal temporal scale-space representation of the temporal test signal at the different temporal scales, possibly complemented by a scale-dependent scaling of the magnitude values, depending on the choice of $L_p$-norm in the wavelet representation and the choice of scale normalization power $\gamma$ in the scale-normalized derivative concept.

#### Finite $L_p$-norms for the temporal derivatives of the time-causal limit kernel

A regularity requirement that one usually imposes on wavelet functions is that they should be in both $L_1({\mathbb {R}})$ and $L_2({\mathbb {R}})$. This property can be easily shown for the temporal derivatives of the time-causal limit kernel, as follows:

Consider a partial fraction decomposition of the Laplace transform (9) of the infinite convolution of truncated exponential kernels that defines the time-causal limit kernel according to (25):

72 $$\begin{aligned} H_{\varPsi }(q;\; \tau , c) = \prod _{k=1}^{\infty } \frac{1}{1 + \mu _k q} = \sum _{k=1}^{\infty } \frac{A_k}{1 + \mu _k q}, \end{aligned}$$

with $\mu _k$ as functions of $\tau$ and *c* according to (13) and (14), and where the coefficients $A_k$ can be determined by first multiplying both sides of the equation by $(1 + \mu _k q)$ and then setting $q = -1/\mu _k$, leading to

73 $$\begin{aligned} A_k = \prod _{i=1, i \ne k}^{\infty } \frac{1}{1 - \frac{\mu _i}{\mu _k}}. \end{aligned}$$

Interpreted over the original temporal domain, this means that the time-causal limit kernel can be written in terms of the following decomposition as a sum of truncated exponential functions:

74 $$\begin{aligned} \varPsi (t;\; \tau , c) = \sum _{k = 1}^{\infty } A_k \, h_{\text{ exp }}(t;\; \mu _k) = \sum _{k = 1}^{\infty } \frac{A_k}{\mu _k} \, e^{-t/\mu _k} \quad (t \ge 0). \end{aligned}$$

Thus, the *n*:th order temporal derivative of the time-causal limit kernel will have the following series representation:

75 $$\begin{aligned} (\partial _{t^n} \varPsi )(t;\; \tau , c) = \sum _{k = 1}^{\infty } \left( \frac{-1}{\mu _k} \right) ^n \frac{A_k}{\mu _k} \, e^{-t/\mu _k} \quad (t \ge 0). \end{aligned}$$

When time *t* tends to infinity, this function will in the limit tend towards zero, and as fast as exponentially with respect to he slowest time constant $\mu _1$. Since $(\partial _{t^n} \varPsi )(t;\; \tau , c)$ is additionally finite for finite values of *t*, it follows that both the $L_1$- and the $L_2$-norms of $\partial _{t^n} \varPsi$ will be finite, implying that $\partial _{t^n} \varPsi \in L_1(R) \cap L_2(R)$, thus proving the result.

#### Time-causal and time-recursive wavelets for real-time and time-critical applications

These resulting wavelets described in this section, consisting of temporal derivatives of the time-causal limit kernel, will be completely time-causal. The convolutions^13^ between these wavelet kernels and a temporal measurement function can also be computed in a completely time-recursive way, thus eliminating the need for additional temporal buffering and in turn allowing for minimal temporal response times in a time-critical context. In these respects, the temporal derivatives of the time-causal limit kernel may thus have interesting potential use for wavelet analysis with regard to applications that are to be performed over time-causal and time-recursive temporal domains, such as for real-time signal analysis systems, or when modelling physical or biological systems for which access to the relative future in relation to any time moment is not possible.

Another type of time-causal wavelet representation has been proposed and studied by Szu et al. (1992), based on linear combinations of sine and cosine waves multiplied by a truncated exponential function. In this context, the wavelets based on temporal derivatives of the time-causal limit kernel have the conceptual advantage that they are solely based on truncated exponential kernels coupled in cascade, and can therefore be implemented in a fully time-recursive manner.^14^ Additionally, with regard to the discrete implementation of such temporal receptive fields in terms of recursive filters coupled in cascade (according to Sect. 4.2), the computation of wavelets based on temporal derivatives of the time-causal limit kernel, an additional temporal scale level can be computed with just the addition of a single recursive filter, complemented with a discrete temporal difference operator (according to Sect. 5.5).

**Fig. 11:**
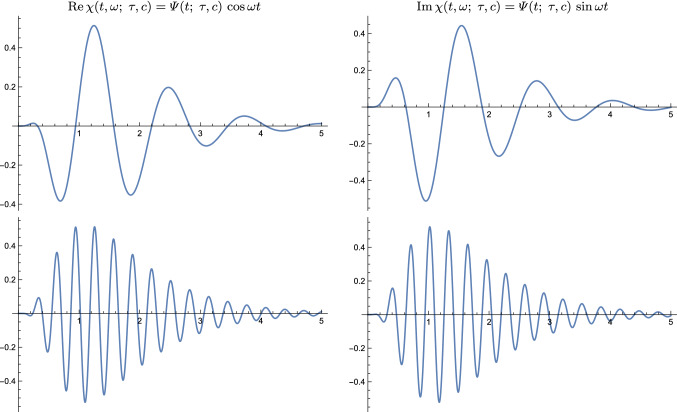
Graphs of the complex-valued extension $\chi (t, \omega ;\; \tau , c) = \varPsi (t;\; \tau , c) \, e^{i \omega t}$ of the time-causal limit kernel $\varPsi (t;\; \tau , c)$ for temporal scale $\tau = 1$ with distribution parameter $c = 2$ and different values of the angular frequency $\omega$. (left column) The real component, corresponding to the time-causal limit kernel multiplied by a cosine wave. (right column) The imaginary component, corresponding to the time-causal limit kernel multiplied by a sine wave. (top row) Angular frequency $\omega = 5$. (bottom row) Angular frequency $\omega = 20$. (Horizontal axes: time. Vertical axes: kernel values)

### Relations to time-frequency analysis

If we combine the time-causal limit kernel $\varPsi (t;\; \tau , c)$ defined according to (25) with pointwise multiplication by a complex exponential function $e^{i\omega t}$, then we obtain a straightforward way of defining a time-causal time-frequency representation of a temporal signal *f*(*t*) according to

76 $$\begin{aligned} S(\omega ;\; \tau , c) = \int \limits _{u=0}^{\infty } f(t-u) \, \varPsi (u;\; \tau , c) \, e^{i \omega u} \, \textrm{d}u, \end{aligned}$$

where the complex-valued extension of the time-causal limit kernel

77 $$\begin{aligned} \chi (t, \omega ;\; \tau , c) = \varPsi (t;\; \tau , c) \, e^{i \omega t} \end{aligned}$$

can be seen as a time-causal analogue^15^ of the Gabor function (Gabor 1946), with the role of the Gaussian kernel $g(t;\; \sigma )$ in the Gabor function

78 $$\begin{aligned} G(t, \omega ;\; \sigma ) = g(t;\; \sigma ) \, e^{i \omega t} = \frac{1}{\sqrt{2 \pi } \, \sigma } e^{-t^2/2\sigma ^2} \, e^{i \omega t} \end{aligned}$$

now replaced the by the time-causal limit kernel $\varPsi (t;\; \tau , c)$ for $\tau = \sigma ^2$. Figure 11 shows graphs of a few examples of such complex-valued extensions of the time-causal limit kernel for different values of the angular frequency $\omega$ in relation to a given temporal scale $\tau$.

In this context, the time-causal limit kernel serves as a temporal window function for computing a windowed Fourier transform, to give better localization properties in the temporal domain compared to a regular Fourier transform, and where the window function in this case, in contrast to the more common choice of a Gaussian window function, is fully time-causal, to allow for real-time processing as well as realistic modelling of real-world physical and biological processes, where access to the relative future in relation to any time moment is simply not possible.

#### Relations to the Gammatone filter

The complex-valued extension of the time-causal limit kernel in (77) is specifically closely related to the Gammatone filter (Johannesma 1972; Patterson et al. 1987, 1995; Hewitt and Meddis 1994) in auditory processing

79 $$\begin{aligned} \gamma (t) = a \, t^{n-1} e^{-2\pi b t} \cos (2\pi \phi \, t + \alpha ), \end{aligned}$$

with the main difference being that the truncated exponential kernels used in this auditory filter have equal time constants, and can thus under a convolution operation be composed into a single monomial multiplied by the complex exponential, in analogy with Eq. (19), and thereby corresponding to a uniform distribution of the temporal scale levels according to Sect. 2.5, whereas the temporal scale levels in the complex-valued extension of the time-causal limit kernel are constructed according to a geometric distribution of the temporal scale levels according to Sect. 2.3, thus, in turn, allowing for different and more rapid temporal dynamics.

Another minor difference is that the phase of the Gammatone filter is represented as a phase angle $\alpha$ of a cosine function, whereas the phase of the complex-valued extension of the time-causal limit kernel is represented as the phase value of a complex exponential.

#### Relations to the Heisenberg group

The time-frequency representation defined according to (76) has the theoretically attractive property that it is closed under (i) translations over time, (ii) multiplicative shifts in the frequency of periodic or repetitive temporal signals and (iii) uniform scaling transformations of the temporal axis with discrete scaling factors *S* that are integer powers of the distribution parameter *c*. Hence, except for the necessary discretization of the temporal scale parameter according to a geometric distribution, which implies closedness over a discrete set of scaling factors as opposed to as over a continuum, this time-frequency representation has the ability to capture similar types of transformations of the signal as the Gabor family, and as can be modelled by the Heisenberg group, see (Feichtinger and Gröchenig 1992). In this way, the complex-valued time-causal limit kernel provides a way to define a scale-covariant time-frequency representation also over a time-causal temporal domain.

#### Extension to an additionally time-recursive time-frequency transform

If one additionally wants these time-frequency representations to also be time recursive, then it is possible to modify this construction slightly, by instead multiplying the input signal by a set of complex exponentials and then filtering the resulting complex-valued signal with the time-causal limit kernel (according to Eq. 81), thus implying that this time-frequency transform can be implemented discretely in terms of a set of recursive filters that operate over time on the pointwise multiplication of the input signal with a set of complex exponential functions. The difference will then be that the phase values will have to be compensated *a posteriori*, whereas the magnitude values of the corresponding spectrogram will be preserved. An earlier version^16^ of this type of theoretical model has been successfully used for computing auditory receptive fields (Lindeberg and Friberg 2015a, b), as will be further described in Sect. 7.2.

## Applications to modelling temporal variations in biological systems

In this section, we will describe different application domains of using the theory for temporal scale-space representation, specifically the time-causal limit kernel, to model temporal variations in biological signals.

### Temporal basis functions in spatio-temporal receptive field models

In (Lindeberg 2011, 2013a), a general model for spatio-temporal receptive fields is derived of the form

80 $$\begin{aligned} T(x_1, x_2, t;\; s, \tau ;\; v, \varSigma )= & {} g(x_1 - v_1 t, x_2 - v_2 t;\; s, \varSigma )\,\nonumber \\{} & {} h(t;\; \tau ) \end{aligned}$$

where

- $x = (x_1, x_2)^T$ denotes the image coordinates,
- *t* denotes time,
- *s* denotes the spatial scale,
- $\tau$ denotes the temporal scale,
- $v = (v_1, v_2)^T$ denotes a local image velocity,
- $\varSigma$ denotes a spatial covariance matrix determining the spatial shape of an affine Gaussian kernel $g(x;\; s, \varSigma ) = \frac{1}{2 \pi s \sqrt{\det \varSigma }} e^{-x^T \varSigma ^{-1} x/2s}$,
- $g(x_1 - v_1 t, x_2 - v_2 t;\; s, \varSigma )$ denotes a spatial affine Gaussian kernel that moves with image velocity $v = (v_1, v_2)$ in space-time and
- $h(t;\; \tau )$ is a temporal smoothing kernel over time.

This model for zero-order spatio-temporal receptive fields should, in turn, be complemented by spatial and temporal differentiation to lead to spatio-temporal receptive fields with positive and negative lobes that are balanced in the sense of the integral of the filter weights being equal to zero.

In (Lindeberg 2016, 2018a, 2021b), it is described how the time-causal limit kernel can be successfully be used as the temporal smoothing kernel in this context, *i.e.*, $h(t;\; \tau ) = \varPsi (t;\; \tau )$ with $\varPsi$ defined from its Fourier transform according to (25), and allowing for truly time-causal and time-recursive model of spatio-temporal receptive fields, which in turn enable provable scale covariance and scale invariance properties over the temporal domain.

By comparisons with biological visual receptive fields measured by electrophysiological cell recordings by DeAngelis et al. (1995), DeAngelis and Anzai (2004), it is shown in (Lindeberg 2016, 2021b) that this spatio-temporal receptive field model very well captures the qualitative shape of lagged and non-lagged LGN neurons as well as simple cells in the primary visual cortex (V1).

### Temporal basis functions in spectro-temporal receptive field models

In (Lindeberg and Friberg 2015a, b), a theoretical framework for idealized models of auditory receptive fields is presented, based on a two-stage model consisting of time-causal spectrograms followed by spectro-temporal receptive fields applied on these, and which comprises covariance and invariance properties under natural sound transformations, such as frequency shifts and glissando transformations.

The time-causal spectrograms in this model are defined according to

81 $$\begin{aligned} S_h(t, \omega ;\; \mu ) = \int \limits _{t'=-\infty }^{\infty } h_\textrm{composed}(t - t';\; \mu ) \, f(t') \, e^{-i\omega t'} \, \textrm{d}t', \end{aligned}$$

where the temporal integration kernel $h_\textrm{composed}$ is from theoretical arguments constrained to be the convolution of a set of truncated exponential kernels coupled in cascade. Following the arguments in this paper, and further restricting this kernel to be a time-causal limit kernel $\varPsi$, we can extend the previous theoretical framework for multi-scale spectrograms to also comprise temporal scale covariance.

In the second-stage model of spectro-temporal receptive fields in this theory, the idealized form of auditory receptive fields are from theoretical arguments constrained to be of the form

82 $$\begin{aligned} A(t, \nu ;\; \varSigma ) = \partial _{t^{\alpha }} \partial _{\nu ^{\beta }} \left( g(\nu - v t;\; s) \, T(t;\; \tau _a) \right) \end{aligned}$$

where

- $\partial _{t^{\alpha }}$ represents a *temporal derivative operator* of order $\alpha$ with respect to time *t* which could alternatively be replaced by a glissando-adapted temporal derivative of the form $\partial _{\overline{t}} = \partial _t + v \, \partial _{\nu }$,
- $\partial _{\nu ^{\beta }}$ represents a *logspectral derivative operator* of order $\beta$ with respect to logarithmic frequency $\nu$,
- $T(t;\; \tau _a)$ represents a *temporal smoothing kernel* with temporal scale parameter $\tau _a$, which should in the time-causal case be a set of truncated exponential kernels coupled in cascade,
- $g(\nu - v t;\; s)$ represents a Gaussian *spectral smoothing kernel* over logarithmic frequencies *v* with logspectral scale parameter *s* and *v* representing a glissando parameter making it possible to adapt the receptive fields to variations in frequency $\nu ' = \nu + v t$ over time.

By comparison with biological auditory receptive fields measured by electrophysiological cell recordings by Qiu et al. (2003), Andoni et al. (2007), Machens et al. (2004), Elhilali et al. (2007) and Atencio and Schreiner (2012), it is shown in (Lindeberg and Friberg 2015a) that the idealized receptive fields from this model agree qualitatively very well with biological auditory receptive fields measured in the inferior colliculus (ICC) and primary auditory cortex (A1) of mammals.

By following the arguments regarding temporal smoothing in this paper, and constraining the temporal kernel in the above model to be a time-causal limit kernel, $T(t;\; \tau _a) = \varPsi (t;\; \tau _a)$, it follows that the auditory covariance properties in the spectro-temporal receptive field model can be extended to also comprise temporal scale covariance.

### Temporal scales in neural signals

In this section, we describe previous evidence and use of multiple temporal scales in neural signals, with relations to the theory for processing temporal signals at multiple scales presented in this paper.

Concerning the use of multiple temporal scales for processing neural signals, Goldman (2009) shows how neural responses can be maintained by a purely feedforward mechanism, which thus implements a temporal memory. In his model, a set of first-order integrators with equal time constants is used. By instead using different time constants of the first-order integrators, as used for the implementation of the time-causal limit kernel, we can get a more compact model for the memory buffers, requiring less wetware or computational modules, with the additional benefit that the time constants obey a self-similar logarithmic distribution.

Tsao et al. (2018) show how temporal information in the lateral entorhinal cortex is robustly encoded over a wide range of temporal scales, from time scales of seconds to hours, where specifically the brain handles multiple scales in parallel, consistent with the underlying construction of a multi-scale representation over the temporal domain, and specifically using a multi-scale temporal representation as a temporal memory. In a further study of the primate entorhinal cortex, Bright et al. (2020) experimentally model time cells in this brain area as single truncated exponentials, in line with theoretical model in Eq. (16), although also complemented with a Gaussian smoothing step that leads to the ex-Gaussian model, and conclude that the time cells in the entorhinal cortex use a spectrum of time constants to construct a temporal record of the past in support of episodic memory. In a study of cerebellar unipolar brush cells, Guo et al. (2021) show that the population of neurons generates a continuum of multi-scale temporal representations, with essentially a logarithmic distribution of the temporal scale levels, consistent with the distribution of temporal scale levels used for the temporal scale-space representation and its associated temporal memory model based on the time-causal limit kernel.

In their computational model, of temporal memory, Howard and Hasselmo (2020) propose that time cells in the hippocampus can be understood as a compressed estimate of events as a function of the past, and that temporal context cells in the entorhinal cortex can be understood as the (real-valued) Laplace transform of that function, respectively, where the Laplace transform in turn arises from the integration with truncated exponential kernels with different time constants, as are used as the unique primitive time-causal temporal smoothing kernel that are guaranteed to not increase the number of local extrema or zero-crossings in the signal. Howard (2021) gives a more general overview of mechanisms for temporal memory, including the use of multiple first-order temporal integrators as arising from this theory.

In an fMRI study of memory recall in human subjects over large variations in the time elapsed after the event, Monsa et al. (2020) conclude that scale-selective activity characterizes autobiographical memory processing and may provide a basis for understanding how the human brain processes and integrates experiences across temporal scales in a hierarchical manner.

Holcombe (2009) gives a general overview of different temporal scale limits in visual perception, in particular describing a distinction into slow and fast temporal processes, which are hypothesized to originate from neural processes over different ranges of temporal scales. In an fMRI study of the human ventral stream, Gauthier et al. (2012) show that the widths of temporal integration windows increase at higher hierarchical levels in the visual hierarchy.

Regarding the use of multiple temporal scales in auditory perception, Atencio and Schreiner (2012) show examples of spectro-temporal receptive fields in the primary auditory cortex (A1) with different spectro-temporal scale characteristics; broadly tuned receptive fields with short temporal duration and narrowly tuned receptive fields with longer temporal duration. Chait et al. (2015) investigate how different temporal scales interact in speech perception and suggest that human speech perception uses multi-time resolution processing. Teng et al. (2016) provide evidence that the auditory system extracts fine-detail acoustic information using short temporal windows and uses long temporal windows to abstract global acoustic patterns. Concerning the specific area of birdsong, Gentner (2008) shows how the use of multiple temporal scales within the acoustic pattern hierarchy conveys information about the individual identity of the singer. Osman et al. (2018) also propose a hierarchy of temporal scales for discriminating and classifying the temporal shapes of sound in different auditory cortical areas.

In a wider study regarding the visual, somatosensory and auditory cortices, Latimer et al. (2019) found that the behaviour of the adaptive responses that they observe can be accounted for by fixed filters that operate over multiple time scales. By developing a method for estimating temporal scales in neuronal dynamics, Spitmaan et al. (2020) found that most neurons exhibited multiple temporal scales in their response, which consistently increased from parietal to prefrontal and cingulate cortex. Miri et al. (2022) in turn suggest that gaze control requires integration over distributed temporal scales.

We propose that if the aim is to build mathematical models of such neural, perceptual or memory processes, then the mathematical theory for time-causal scale-space kernels presented in this paper should be ideally suited for building such models that are both time-causal and time-recursive. Specifically, if the aim is to build such temporal models that can handle multiple temporal scales in a way that respects temporal scale covariance, and under an architectural setting that corresponds to multiple primitive temporal smoothing stages coupled in cascade, then the time-causal limit kernel (described in Sect. 3.1) with its temporal derivatives (described in Sect. 5) constitutes a canonical class of temporal basis functions to be used in such models.

As a consequence of the temporal delay of such time-causal kernels (Eqs. 10 and 39), any time-causal perceptual process will be associated with an inherent temporal delay (complemented with the processing time of the neural processes that implement the corresponding computations), implying that the representation of the present (White 2020) will in practice be a representation of some (temporally extended) temporal moment(s)^17^ in the past, unless complemented with extrapolation/prediction (White 2018) over a time period corresponding to the temporal delay(s) of the perceptual process that lead to that percept. Still, however, a representation of the present, with or without temporal prediction implying without or with an inherent temporal delay, will by necessity be a representation of a temporally “fuzzy” present.

In their review of the use of multiple temporal scales in the brain, Cavanagh et al. 2020 state that short temporal windows facilitate adaptive responding in dynamic environments, whereas longer temporal windows promote the gradual integration of information across time, and specifically concerning the notion of multiple temporal scales they conclude a heterogeneity of temporal receptive fields at the level of single neurons within a cortical region, consistent with the aims behind the theory for temporal scale-space representation described in this article.

## Implications of the presented theory with regard to the philosophy of time and perceptual agents

The subject of this paper has been to describe a theoretical framework for handling the notions of time and temporal scales for a perceptual system or a neural system, in a both principled and theoretically well-founded manner. Since this subject has implications regarding how we consider the notion of time for a perceptual agent, we will in this section describe relations to the philosophy of time (Mölder et al. 2016; Callender 2017), which is still an open topic in the area of philosophy.

The notion of time is something that we usually take for granted. Still there is no fully established definition for this concept. Already St. Augustine (354–430) stated (Outler, transl. 1955, Book 11, page 193):

What, then, is time? If no one asks me, I know what it is. If I wish to explain it to him who asks me, I do not know. Yet I say with confidence that I know that if nothing passed away, there would be no past time; and if nothing were still coming, there would be no future time; and if there were nothing at all, there would be no present time.

According to Newtonian or Galilean space-time, we can treat time as flowing continuously and define a universally valid notion of global time. According to Einstein’s relativity theory (1905, 1916), different observers can measure time differently, being affected by the relative velocity between the observers. Thus, measurement of time is a local property (attached to the path that an observer or a clock follows in space-time), and (at very high relative velocities) different observers may not even be able to agree on the temporal ordering between different temporal events in the world.^18^^,^^19^^,^^20^ This treatment deals with the handling of time for a single perceptual agent that observes a dynamic world using time-causal receptive fields as temporal primitives in its perceptual system.

Originating from a paper by McTaggart (1908), there are two main theories regarding time in the area of philosophy: According to the A-theory, A-series events are ordered by which are present, which are past, and which are future (tensed propositions), whereas according to B-theory, B-series events are ordered by which come before and which come after (tenseless propositions) (Zalta (ed.), Stanford Encyclopedia of Philosophy 2020). Thus, A-theory is closer to how we perceive time as humans (and similar to St. Augustine’s view above), whereas B-theory is closer to how we describe temporal phenomena in physical theories of the world.

In a treatment about the notion of temporal presence, Power (2016) discusses how we are able to maintain a perception of changes in the world in our representation of the present. Essentially using the argument that the temporal present is an instantaneous property (valid at a single time moment only), while arguing that the perception of changes requires access to properties of the world over an extended temporal interval, he concludes that A-theory is false, since extended temporal properties cannot exist in a representation of the temporal presence at a single time moment.^21^

From the viewpoint of a temporal multi-scale analysis as developed in this paper, where each measurement of properties in the world requires integration over a non-infinitesimal temporal interval, it does, however, follow that any perceptual measurement of the world will have to be performed at some non-infinitesimal inner temporal scale, and thus correspond to integration over a non-infinitesimal duration over time. From such a viewpoint there is no contradiction relative to a *perceptual representation of the present*, since a multi-scale representation of the present will always occur over multiple temporal scales, and will thus have the possibility to collect information about how properties in the world change over time over extended temporal intervals.

Additionally, in human perception, there are dedicated perceptual mechanisms for registering changes or motion over time;^22^ compare, for example, with the illusion of the motion after effect (Wohlgemuth 1911), implying that if you look out of a window of a moving train for a long time, and if the train suddenly stops, you may for a while perceive a (physically non-existent) motion in the opposite direction. Alternatively, you may encounter a similar illusion if looking at the motion of streaming water for a sufficiently long time, and then perceive motion in the opposite direction if you change your viewing direction to focus on a static object. There are also static stimuli that give rise to perception of motion (see, e.g. Conway et al. 2005).

**Fig. 12:**
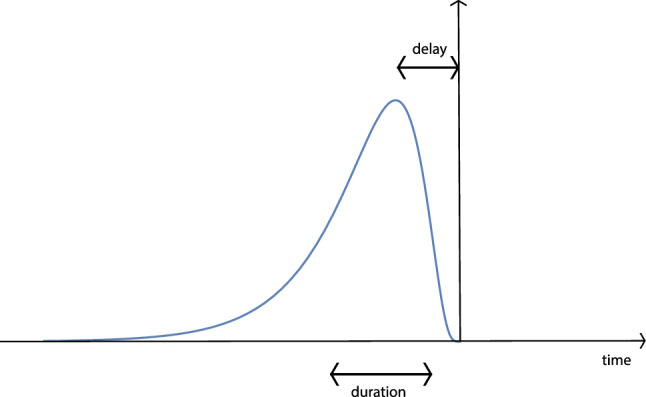
Illustration of non-infinitesimal temporal duration of any physical measurement that arises as a consequence of a non-infinitesimal inner temporal scale in a physical temporal measurement device, specifically for any biological sensory or perceptual system, as well as the nonzero temporal delay of any time-causal temporal receptive field, which implies that the representation at any present moment will *de facto* instead be a representation of what happened some amount of time ago in the past. For the scale-covariant time-causal limit kernel proposed as the most idealized model of a temporal receptive field in this article, the temporal delay will specifically be proportional to the temporal scale measured in units of $[\text{ time}]$, thus implying longer temporal delays at coarser temporal scales. (For a physical or biological implementation of these notions, there will also be another complementary temporal delay, not treated further here, caused by the time it takes to carry out the actual computations in the perceptual system.) (The vertical arrow in this illustration is intended to represent the present moment. The blue curve, in turn, reflects how different information from different temporal moments in the past contribute to the representation of the present at that present moment. To represent the temporal duration of the time-causal temporal smoothing kernel, we have in this illustration drawn the “full width half maximum” (FWHM), which is proportional to the temporal standard deviation of the temporal scale-space kernel, in other words proportional to the square root of the temporal scale parameter $\tau$)

The model for temporal multi-scale processing developed in this paper does thus make the following assumptions concerning the handling of the notion of time for a perceiving agent: The perceptual system of the perceiving agent has a lowest layer of biophysical sensors, which performs temporal integration of the underlying physical signal with some shortest time constant corresponding to the smallest possible inner temporal scale of the perceiving agent. Then, successive layers of such operations are coupled in cascade in a hierarchical manner over that first layer, leading to a layered architecture in the perception system, with successively longer effective time constants at higher layers corresponding to coarser temporal scales. Each such representation in any layer of the hierarchy operates on input information acquired in the present, possibly complemented with access to memory buffers of the past. Thus, from the perspective of the perceiving agent, he or she cannot have any access to the actual physical present in the external world (“das Ding an sich”; Kant 1783, 1902), but instead just access to a temporally blurred representation of the present, which from the perspective of the perceiving agent is the *only* available representation of the present^23^ (see Fig. 12).

From the representation of the (temporally blurred fuzzy) present, the internal perceiving system of the agent may also compute representations at coarser temporal scales, which by the temporal delays inherent to the time-causal temporal processes will also serve as a temporal memories of the past. The perceiving agent has no access to a video or audio recording of the past. Instead, the only possible representation of the past is what is stored in the temporal memories of the perceiving agent.^24^ Some of these memories may be of a short term nature and soon be overwritten by more recent information, while other memories may be stored for further longer term access.

A more technical problem in relation to temporal memory concerns making estimates of the duration of a temporal event. According to the standard methodology in physics, one would use a clock, register the times of the beginning and the end of the temporal event and compute the duration from the difference between these temporal moments (a B-series type of measurement). A biological perceiving agent does, however, not have access to any explicit clock, and there is no evidence for an accurate inner clock in the human brain that a human perceiving agent could relate to for directly measuring the duration of temporal events (Wittmann 2009).

From the viewpoint of a temporal multi-scale analysis, it is, however, in principle possible to estimate the duration of a temporal event by operating on representations at multiple temporal scales and comparing the relative strengths of their responses, thus using A-type measurements in the (time-delayed) present as opposed to quantitative B-type temporal relations for estimating temporal duration. In (Lindeberg 2018a), it is shown how it is possible to define multi-scale spatio-temporal visual operations that respond by their strongest response over temporal scales at a temporal scale corresponding to the temporal duration of the temporal event, thus estimating the duration of a temporal event based on measurements at a single temporal moment only, although a very special temporal moment at which the response assumes extrema over both time and temporal scales. This is an extension of spatial scale selection (Lindeberg 1998a, 2021a), which makes it possible to estimate spatial scales without need for explicitly laying out a ruler.^25^

Due to the temporal delays of the time-causal receptive fields that drive this perceptual engine over time, any representation of the present will not be a representation of the actual present moment, but instead of what had occurred at some temporal moments (or rather temporal intervals) in the past. Furthermore, representations at coarser temporal scales will harbour the traces of events that occurred further in the past compared to representations at finer scales, thus providing basic mechanisms for temporal memory buffers.

**Fig. 13:**
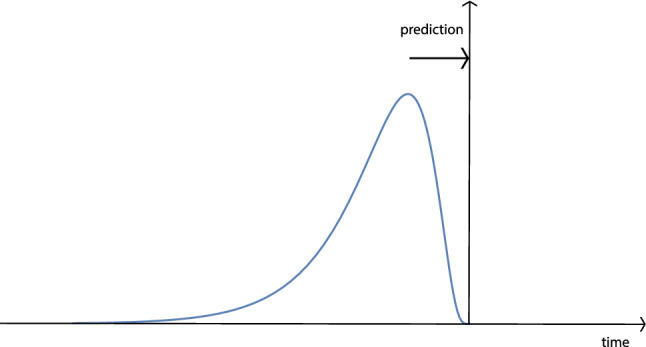
The temporal delays of the time-causal receptive fields resulting from the presented theory call for a mechanism for performing temporal prediction to extrapolate the *de facto* time-delayed representation of the present (here represented as the temporal peak of the temporal receptive field marked in blue) to a better representation of the actual present (here represented by the vertical line on the time axis), to enable better temporal dynamics for a perceptual agent that interacts with a dynamic world. (Additionally, it is, of course, for other purposes also preferably to also attempt to perform predictions into the actual future in relation to any time moment, to enable temporal planning and to compensate for the time it will take to execute the actions called for by the perceptual agent. The latter types of temporal predictions are, however, not assumed to influence the representation of the present in this treatment)

To make it possible for the perceiving agent to handle fast occurring temporal events in a dynamic world, it is therefore extremely valuable for a perceiving agent to be able to perform predictions from the time-delayed perceptual present to at least the actual physical present, so as to be able to coordinate his or her actions with fast occurring temporal phenomena (Fig. 13). Given that it will additionally take time to plan and execute an action in practice, it is in a similar way essential that the perceptual agent can perform predictions into the actual future in relation to the actual present moment when planning and executing an event. Even further predictions to the future may of course also be valuable for longer term planning, and to be able to make such longer term predictions, it is very valuable to have an explicit memory of the past over longer temporal scales. Thus, the notion of multiple temporal scales is also important for making predictions into the future, for different time scales into the future.

For the brain of a perceiving agent, its ability to predict what will happen in the future may therefore be one of the most critical factors that determine its ability to survive and reproduce in a competition between individuals and species in the survival of the fittest (Darwin 1859, 2004; Spencer 1864, 2020). Minimizing the prediction error, has been proposed as main principle underlying brain function (Friston 2010; McCrone 2022). It has also been argued that the sensory cortex is optimized for prediction of future input (Singer et al. 2018), and furthermore been demonstrated that it is possible to learn the receptive fields of deep neural networks by training the networks to predict the relative future from pre-recorded video sequences of natural scenes (Singer et al. 2018; Kwon and Park 2019; Lotter et al. 2020). Low-level neuronal learning mechanisms have also been proposed in terms of predicting future activity (Luczak et al. 2022).

To conclude, we argue that in a A-theory type treatment of time for a perceptual agent, it is essential to complement previous such treatments with explicit notions of (i) non-infinitesimal temporal scales for *any* representations of the present, and also to incorporate (ii) the unavoidable temporal delays of time-causal receptive fields that determine the functional properties of perceptual systems. In a corresponding manner, given the extended temporal delays of even the fastest temporal processes in, e.g. human vision, it is essential to complement the perceptual process with (iii) mechanisms for temporal predictions, since otherwise the actions of the perceiving agent will be too slow to be able to handle and cope with rapid temporal phenomena in the environment.^26^, These three notions are immediate consequences of treating temporal perception as a consequence of a temporal measurement problem, where information in physical stimuli has to be integrated over non-infinitesimal durations over time (a main assumption underlying the formulation of the presented temporal scale-space theory), and making a notion such as the instantaneous present *de facto* impossible for a perceptual agent.^27^

Given the working hypothesis that perception has to involve some mechanisms for temporal prediction to compensate for the non-avoidable temporal delays associated with time-causal temporal integration over non-infinitesimal neighbourhoods over time, our conscious experience of the present in the world, thus has to *synthesize* a view of the world, created by our brain, and truly corresponding to “controlled hallucination” (Koenderink 2011; Clark 2016; Paolucci 2021; Seth 2021). It is a “hallucination” in the sense that the view of the present is not actually a view of how the world is or was at the moment that it was first registered and then passed on to further processing. It is on the other hand “controlled” in the sense that it is grounded on biophysical measurements of properties in the world, and processed by a biological system that has been refined over evolution over a very large number of generations.

Let us finally emphasize that this treatment does not make any claim of being able to judge about the properties of time itself, which can only be made by physical experiments, possibly complemented by theoretical modelling and analysis, as done in the area of theoretical physics. Instead, the treatment in this section concerns how the notion of time is handled by a perceptual agent, specifically how the notion of multiple temporal scales with their associated temporal delays have to be considered in such a context, with a set of immediate implications thereof.

Let us also stress that the model used as basis for this treatment is continuous in time, whereas for a biological neural system that communicates with spikes between its neurons, the underlying communication channels are in reality discrete, however, here assumed to be operating at a temporal scale below the inner temporal scale of the functional processes in the perceptual system.

## Historical developments of temporal scale-space theory

For the reader interested in a historical overview of previous developments of temporal scale-space theory, this section gives an overview of some the main previous contributions in this area that this paper is based on, follows and extends.

Koenderink (1988) pioneered the area of the temporal scale-space representation by proposing his scale-time model based on applying Gaussian smoothing over a logarithmically transformed temporal domain.

A complete classification of the general class of continuous scale-space kernels was first given in (Lindeberg 1993b). While this classification also included the truncated exponential kernels used as main temporal primitives in this paper, the main topic of that book was spatial computer vision, and the specific detailed structure of time-causal scale-space kernels was at first developed further in the more dedicated treatment in (Lindeberg and Fagerström 1996) aimed at video processing, specifically including the logarithmic distribution of the temporal scale parameter in the set of temporal scale channels.

The topic of temporal scale selection was first addressed in (Lindeberg 1997a), including detailed investigations of the response properties of time-causal receptive fields over temporal scales and time, and illustrating how a closely related temporal model based on the time-causal Poisson kernel, in turn assuming a semi-group property over temporal scales, can also serve as a temporal memory of the past.

In (Lindeberg 1997b, 2001) the time-causal model based on the temporal Poisson kernel, specifically the temporal derivatives of this kernel, was used for modelling the temporal variability in biological spatio-temporal receptive fields. In ter Haar Romeny et al. (2001) the temporal variability in biological spatio-temporal receptive fields was modelled using temporal derivatives of Koenderink’s scale-time kernels.

Other temporal scale-space models based on a semi-group property over temporal scales were then studied in (Fagerström 2005, 2007) and (Lindeberg 2011).

In (Lindeberg 2016) a substantial theoretical extension was made of the temporal model based on truncated exponential kernels coupled in cascade, by deriving the time-causal limit kernel, which allows for temporal scale covariance. In (Lindeberg 2017) this model was extended to temporal scale selection, including detailed studies of the temporal response properties and scale selection properties for the cases of a uniform sampling *vs.* a logarithmic sampling of the temporal scale parameter. A general proof was also presented, explaining how previous temporal models based on the assumption of a semi-group property over temporal scales lead to poor temporal dynamics, specifically undesirably long temporal delays.

In (Lindeberg 2016) the developments of the time-causal limit kernel were performed in the context of video processing, and were used for deriving theoretical models of spatio-temporal receptive fields with close relations to biological receptive fields in the lateral geniculate nucleus (LGN) and the primary visual cortex (V1). In (Lindeberg 2018a) this theoretical framework for spatio-temporal receptive fields was extended to scale-covariant spatio-temporal feature detection with integrated spatio-temporal scale selection. In (Lindeberg 2018b) corresponding extensions were made for dense temporal scale selection as well as dense spatio-temporal scale selection. In (Jansson and Lindeberg 2018) a specific application to video analysis was developed to analyze dynamic textures in a temporally scale-covariant manner. In (Lindeberg 2021b) the same theoretical model for spatio-temporal receptive fields based on using the time-causal limit kernel and its temporal derivatives as temporal basis functions was used for modelling biological vision in an axiomatic normative theory of visual receptive fields

In (Lindeberg and Friberg 2015b, a) parallel developments were made for auditory signals, showing how main classes of time-frequency transforms (spectrograms) can be derived in an axiomatic manner, as well as how auditory receptive fields at a higher level can also be axiomatically derived with very close similarities to biological auditory receptive fields.

Most of the previous developments of the temporal scale-space theory relevant for the treatment in this paper have, however, been performed with regard to visual processing, and in the context of models for spatio-temporal receptive fields. Some parallel developments have on the other hand been performed with regard to auditory processing.

Anticipating that this could be a cause to problems for a reader from a background in biology or signal processing, who is interested in analysing or modelling purely temporal phenomena using a corresponding theory, and wanting to get reasonably quickly into the associated concepts, a first main purpose of this article has therefore been to give a dedicated and self-contained treatment that develops the relevant temporal scale-space theory for the specific domain of purely temporal signals, without having the theory intertwined with concepts regarding spatial or frequency domains, as is the case in the previously available literature, dealing with visual or auditory processing.

We do additionally outline extensions of this temporal scale-space theory to forming time-causal and time-recursive wavelet representations as well as time-causal and scale-covariant time-frequency representations, which do both provide novel contributions with regard to these areas.

With regard to modelling of temporal phenomena in biology, we develop detailed comparisons to other purely temporal models that can be used for such purposes, including ways of translating results from those models to models based on the time-causal limit kernel studied in this paper. With regard to such purposes, we do also extensively relate to previous work on modelling temporal scales in neural signals, for which we proposed that the presented temporal scale-space model could provide a both theoretically and practically valuable tool. Specifically, we present a general procedure for fitting the time-causal limit kernel to non-negative data, without any need for making use of an explicit expression of the time-causal limit kernel over the temporal domain.

We do finally present implications of the presented theory to fundamental concept formation in the area of the philosophy of time and regarding non-infinitesimal inner temporal scales for any temporal sensor measurement in a perceptual agent, including the resulting inevitable nonzero temporal delays implied by that, in turn implying a need for making predictions into the real present moment, to be able to handle rapid temporal phenomena in the environment.

## Summary and conclusions

We have presented a theory for how temporal smoothing of temporal signals can be performed in such a way that it guarantees that the smoothing process does not create new artificial structure in the signal, in the sense that the number of local extrema in the signal, or equivalently the number of zero-crossings, is guaranteed to not increase from finer to coarser temporal scales. Additional critical components of this theory are temporal causality, implying that we are not allowed to access information from the future in relation to any time moment, and temporal recursivity, implying that the temporal smoothing process should not require any other temporal memory of the past than the resulting temporal scale-space representations themselves.

A complete classification of the linear and shift-invariant convolution kernels that obey these properties has been given, based on an earlier treatment in (Lindeberg and Fagerström 1996), in turn based on earlier classical results by Schoenberg (1948, 1950). For continuous signals, the corresponding temporal scale-space kernels consist of truncated exponential kernels, corresponding to first-order integrators coupled in cascade, and for discrete signals, first-order recursive filters coupled in cascade (Sect. 2.2).

As a conceptual extension of this general approach, we have described a specific subset of choosing these kernels in such a way that temporal scale covariance is obtained. The corresponding time-causal limit kernel that permits scale covariance, which is a novel construction in (Lindeberg 2016), is the limit case of an infinite number of truncated exponential kernels coupled in cascade, with specific choices of the temporal time constants (Sect. 3.1).

Temporal scale covariance in this context means that if the input signal is rescaled by some uniform temporal scaling factor $S = c^i$, where *c* is the distribution parameter of the time-causal limit kernel and *i* is some integer, then the result of performing temporal smoothing on the rescaled temporal signal is the same as performing temporal smoothing on the input signal, followed by a corresponding rescaling of the processed original signal, and complemented by a shift of *i* units along the scale dimension (Sect. 3.1.3).

These temporal kernels, optionally combined with their temporal derivatives, do in this way constitute a canonical class of temporal basis functions for numerous purposes of temporal modelling, in situations when the temporal operations have to be time-causal and time-recursive, and in addition have the ability to handle temporal information over multiple temporal scales in a theoretically well-founded manner. With appropriate scale normalization of the temporal derivatives, the temporal derivatives of the time-causal limit kernel are also truly scale covariant, with preserved magnitude values of temporal derivatives at matching temporal scale levels under scaling transformations, in turn allowing for truly scale-invariant processing under temporal scaling transformations of the input signal (Sect. 5.3).

We have given an explicit expression for the time-causal limit kernel in the Fourier domain (25) and although the kernel lacks a compact closed-form expression over the temporal domain, we have shown how it can be related to other temporal models, such as Koenderink’s scale-time kernels (Sect. 3.3) and the ex-Gaussian model, which is the convolution with an exponential kernel with a single truncated exponential function (Sect. 3.4). We have also presented a general methodology for how the parameters in a model based on a (temporally either unshifted or time-shifted) time-causal limit kernel can be determined from lower-order temporal moments of some other temporal function or temporal signal (Sect. 3.4.2 and Appendix A.3).

We have described how these kernels can be implemented on discrete data, based on a set of first-order recursive filters coupled in cascade, where also the discrete implementation guarantees that new local extrema, or equivalently new zero-crossings, cannot be created from finer to coarser levels of scale (Sect. 4). The discrete implementation of temporal derivatives is straightforward, in terms of small support finite difference operators applied to the discrete temporal scale-space representation (Sect. 5.5). Thus, the discrete implementation is highly efficient and lends itself to real-time applications.

We propose that the presented theory, serving as a *normative theory of purely temporal receptive fields*, provides a canonical way of defining multi-scale representations of temporal signals in situations where the signal operations have to be truly time-causal, because of lack to access of future information in real-time scenarios, and time-recursive, because of a need to keep memory buffers of the past to a minimum in terms of memory requirements. Specifically, we propose that the time-causal limit kernel with its temporal derivatives constitutes a canonical class of temporal basis functions in situations when the temporal scales may vary, especially when temporal scale covariance and temporal scale invariance are desirable properties.

We have also related the theory to other approaches for processing temporal signals at multiple temporal scales, specifically wavelet analysis and time-frequency analysis. We have outlined how the temporal derivatives of the time-causal limit kernel can serve as time-causal and time-recursive wavelet bases (Sect. 6.1) and how a complex-valued extension of the time-causal limit kernel can be seen as time-causal analogue of Gabor functions, in turn enabling truly scale-covariant time-frequency analysis also over time-causal and time-recursive temporal domains (Sect. 6.2).

Concerning applications of the presented theory, we have described how these time-causal kernels constitute a canonical class of temporal kernels for modelling spatio-temporal and spectro-temporal receptive fields in biological perception (Sects. 7.1–7.2). We have also given a more general overview of the applicability of multiple temporal scale levels in perceptual, memory and cognitive processes in biological nervous systems, as well as given arguments proposing that the time-causal kernels treated in this paper should constitute a corresponding canonical class of temporal kernels when modelling neural signals as well as more general perceptual and temporal memory processes by explicit mathematical models (Sect. 7.3).

Finally, we have presented general arguments for the need for incorporating the notion of non-infinitesimal temporal scales with their associated nonzero temporal delays when considering a perceptual representation of the present (not the same concept as the instantaneous actual present, which a perceptual agent has no possible access to), which then also leads to a direct need for temporal extrapolation or prediction in order to compensate for the temporal delays associated with the time-causal temporal filtering operations in a time-causal perceptual system (Sect. 8). We propose that these arguments should have essential implications for the logical reasoning in A-type theories of time in the philosophy of time, as well as when modelling perceptual agents.

## Figures and Tables

**Fig. 14:**
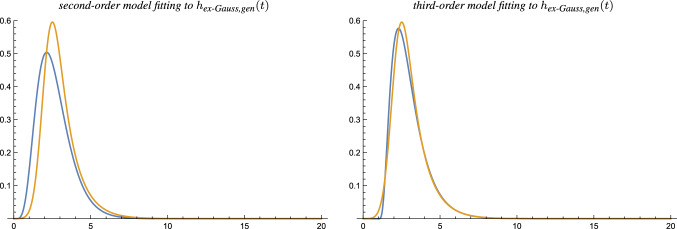
Comparison between (brown curves) the ex-Gaussian model according to (40) and (blue curves) the time-causal limit kernel according (25) approximated using the first $K = 7$ components of the infinite convolution of truncated exponential kernels in cascade. (left) A model with an unshifted time-causal limit kernel fitted using the second-order moment-based method to an ex-Gaussian model with parameters $\mu = 1$, $\sigma = 1/2$, $m = 2$, $a_0 = 0$ and $a_1 = 1$ corresponding to $\tau \approx 1.25$, $c \approx 1.32$, $b_0 = 0$ and $b_1 \approx 1.25$. (right) A model with a time-shifted time-causal limit kernel fitted using the third-order moment-based method to the same ex-Gaussian model with parameters $\mu = 4$, $\sigma = 2$, $m = 2$, $a_0 = 0$ and $a_1 = 1$ corresponding to $\tau \approx 1.25$, $c \approx 1.88$, $t_0 \approx 0.98$, $b_0 = 0$ and $b_1 \approx 1.25$. (Horizontal axes: time. Vertical axes: function values)

## A Appendix A: Relation between the time-causal limit kernel and the ex-Gaussian model used by Bright et al.

In (Bright et al. 2020, see Eqs. 2 and 3), the authors fit a so-called ex-Gaussian model, which is the convolution of an unnormalized Gaussian function with an unnormalized truncated exponential kernel, to the temporal response functions of neurons. With slightly different naming of the variables to avoid notational clashes with the notation used elsewhere in this article, let us consider a temporal response function of the form

83 $$\begin{aligned} h_{\text{ ex-Gauss,gen }}(t) = a_0 + a_1 \int \limits _{u=0}^{\infty } e^{-\frac{(t-m-u)^2}{2 \sigma ^2}} e^{-\frac{u}{\mu }} \, \textrm{d}u, \end{aligned}$$

which after explicit computation of the convolution integral in Mathematica assumes the form

84 $$\begin{aligned}{} & {} h_{\text{ ex-Gauss,gen }}(t) \nonumber \\{} & {} \quad = a_0 + a_1 \sqrt{\frac{\pi }{2}} \, \sigma \, e^{\frac{2 m \mu -2 \mu t+\sigma ^2}{2 \mu ^2}} {\text {erfc}}\left( \frac{m \mu -\mu t+\sigma ^2}{\sqrt{2} \mu \sigma }\right) .\nonumber \\ \end{aligned}$$

### A.1 Second-order moment-based method without flexible temporal offset parameter

In this appendix, we will derive a relation between the above ex-Gaussian model and a corresponding model based on the time-causal limit kernel

85 $$\begin{aligned} h_{\text{ limit-kern,gen }}(t) = b_0 + b_1 \, \varPsi (t;\; \tau , c) , \end{aligned}$$

with the time-causal limit kernel $\varPsi (t;\; \tau , c)$ in (26) defined from its Fourier transform according to (25).

For simplicity, let us first assume that we are in range of the parameter space of the ex-Gaussian model where the temporal delay is small relative to the standard deviation and the time constant $\mu$, such that we do not need to introduce an additional temporal delay in the model (85) based on the time-causal limit kernel. Let us also assume that we can assume that the DC levels in the two models should be equal, such that we can throughout assume that $b_0 = a_0$. Then, our task is to derive a mapping to compute the parameters $b_1$, $\tau$ and *c* in the model based on the time-causal limit kernel from the parameters $a_1$, *m*, $\sigma$ and $\mu$ in the ex-Gaussian model.

The approach that we shall follow is to compute the zero-, first- and second-order temporal moments of the two models with the DC-offsets $a_0$ and $b_0$ suppressed

86 $$\begin{aligned} h_{\text{ ex-Gauss }}(t) = a_1 \sqrt{\frac{\pi }{2}} \, \sigma \, e^{\frac{2 m \mu -2 \mu t+\sigma ^2}{2 \mu ^2}} {\text {erfc}}\left( \frac{m \mu -\mu t+\sigma ^2}{\sqrt{2} \mu \sigma }\right) \end{aligned}$$

and

87 $$\begin{aligned} h_{\text{ limit-kern }}(t) = b_1 \, \varPsi (t;\; \tau , c) , \end{aligned}$$

and determine the mapping between the parameters of the two models from the requirement that the integral, the temporal mean and the temporal variance should be equal.

Computing the (uncentered) temporal moments up to order two of the ex-Gaussian model (86) in Mathematica gives

88 $$\begin{aligned} M_0&= \int \limits _{t = 0}^{\infty } h_{\text{ ex-Gauss }}(t) \, \textrm{d}t \nonumber \\&= a_1\sqrt{\frac{\pi }{2}} \mu \sigma \left( \text {erf}\left( \frac{m}{\sqrt{2} \sigma }\right) \right. \nonumber \\&\left. \quad +e^{\frac{2 m \mu +\sigma ^2}{2 \mu ^2}} {\text {erfc}}\left( \frac{m \mu +\sigma ^2}{\sqrt{2} \mu \sigma }\right) +1\right) , \end{aligned}$$

89 $$\begin{aligned} M_1&= \int \limits _{t = 0}^{\infty } t \, h_{\text{ ex-Gauss }}(t) \, \textrm{d}t \nonumber \\&= a_1 \sqrt{\frac{\pi }{2}} \mu \sigma \left( \mu e^{\frac{2 m \mu +\sigma ^2}{2 \mu ^2}} {\text {erfc}}\left( \frac{m \mu +\sigma ^2}{\sqrt{2} \mu \sigma }\right) \right. \nonumber \\&\quad \left. +(m+\mu ) \left( -{\text {erfc}}\left( \frac{m}{\sqrt{2} \sigma }\right) \right) \right. \nonumber \\&\quad \left. +\sqrt{\frac{2}{\pi }} \sigma e^{-\frac{m^2}{2 \sigma ^2}}+2 (m+\mu )\right) , \end{aligned}$$

90 $$\begin{aligned} M_2&= \int \limits _{t = 0}^{\infty } t^2 \, h_{\text{ ex-Gauss }}(t) \, \textrm{d}t \nonumber \\&= a_1 \sqrt{\frac{\pi }{2}} \mu \sigma \left( -{\text {erfc}}\left( \frac{m}{\sqrt{2} \sigma }\right) \left( m^2+2 m \mu +2 \mu ^2+\sigma ^2\right) \right. \nonumber \\&\quad \left. +2 \mu ^2 e^{\frac{2 m \mu +\sigma ^2}{2 \mu ^2}} {\text {erfc}}\left( \frac{m \mu +\sigma ^2}{\sqrt{2} \mu \sigma }\right) \right. \nonumber \\&\quad \left. +2 \left( m^2+2 m \mu +2 \mu ^2+\sigma ^2\right) \right. \nonumber \\&\quad \left. +\sqrt{\frac{2}{\pi }} \sigma (m+2 \mu ) e^{-\frac{m^2}{2 \sigma ^2}}\right) , \end{aligned}$$

from which we in turn obtain the temporal mean $\delta$ and the temporal variance *V* according to

91 $$\begin{aligned} \delta&= \frac{M_1}{M_0}, \end{aligned}$$

92 $$\begin{aligned} V&= \frac{M_2}{M_0} - \left( \frac{M_1}{M_0} \right) ^2. \end{aligned}$$

#### A.1.1 Method for second-order moment-based model fitting

Using the fact that the temporal mean and the temporal variance of the time-causal limit kernel are given by (Lindeberg 2016, Eqs. 34 and 35)

93 $$\begin{aligned} \delta&= \sqrt{\frac{c+1}{c-1}} \sqrt{\tau }, \end{aligned}$$

94 $$\begin{aligned} V&= \tau , \end{aligned}$$

identifying these expressions and solving for $b_1$, *c* and $\tau$ in the model (87) based on the time-causal limit kernel gives

95 $$\begin{aligned}&b_1 = M_0, \end{aligned}$$

96 $$\begin{aligned}&c = \frac{\delta ^2+V}{\delta ^2-V}, \end{aligned}$$

97 $$\begin{aligned}&\tau = V, \end{aligned}$$

which with $\delta$ and *V* according to (91) and (92) as well as $M_0$, $M_1$ and $M_2$ according to (88), (89) and (90) gives the desired mapping between the ex-Gaussian model (83) and the model (85) based on the time-causal limit kernel.

#### A.1.2 Experimental results

Figure 9 shows examples of ex-Gaussian temporal models approximated by time-causal limit kernels in this way. A conceptual advantage of the time-causal limit kernel in this context, is that we do not need to use or modify a Gaussian kernel to model the initial transient phenomena in a time-causal temporal response function that decays towards zero in an exponential manner towards the tail. In this way, a neural response modelled by the model based time-causal limit kernel would also correspond to a biologically plausible implementation corresponding to temporal integration of the form illustrated in Fig. 2.

### A.2 Extension to a third-order moment-based method involving an additional temporal offset parameter

As a remark concerning extensions, if the ex-Gaussian model is in a range of the parameter space where the temporal delay is large relative to temporal duration of temporal onset of the composed kernel, then an additional temporal offset $t_0$ can be added to the model (85) based on the time-causal limit kernel

98 $$\begin{aligned} h_{\text{ limit-kern,gen }}(t) = b_0 + b_1 \, \varPsi (t-t_0;\; \tau , c) \end{aligned}$$

and an additional computation and identification of the third-order central moments be performed to determine also this parameter in the mapping between the two types of temporal models.

The explicit expression for the unnormalized and uncentered third-order temporal moment of the ex-Gaussian model for $a_0 = 0$ is

99 $$\begin{aligned} M_3&= \int \limits _{t = 0}^{\infty } t^3 \, h_{\text{ ex-Gauss }}(t) \, \textrm{d}t \nonumber \\&= a_1 \sqrt{\frac{\pi }{2}} \sigma \left( \mu {\text {erf}}\left( \frac{m}{\sqrt{2} \sigma }\right) \left( m^3+3 m^2 \mu +6 m \mu ^2+3 m \sigma ^2 \right. \right. \nonumber \\&\left. \left. \quad +6 \mu ^3+3 \mu \sigma ^2\right) \right. \nonumber \\&\left. \quad +\mu e^{-\frac{m^2}{2 \sigma ^2}} \left( -6 \mu ^3 e^{\frac{\left( m \mu +\sigma ^2\right) ^2}{2 \mu ^2 \sigma ^2}} {\text {erf}}\left( \frac{m \mu +\sigma ^2}{\sqrt{2} \mu \sigma }\right) \right. \right. \nonumber \\&\left. \left. \quad +\sqrt{\frac{2}{\pi }} \sigma \left( m^2+3 m \mu +6 \mu ^2+2 \sigma ^2\right) \right. \right. \nonumber \\&\left. \left. \quad +e^{\frac{m^2}{2 \sigma ^2}} \left( m^3+3 m^2 \mu +6 m \mu ^2+3 m \sigma ^2 \right. \right. \right. \nonumber \\&\left. \left. \left. \quad +6 \mu ^3+3 \mu \sigma ^2\right) \right. \right. \nonumber \\&\left. \left. \quad +6 \mu ^3 e^{\frac{\left( m \mu +\sigma ^2\right) ^2}{2 \mu ^2 \sigma ^2}}\right) \right) , \end{aligned}$$

whereas the expression for the normalized and centered third-order moment of the time-causal limit kernel is (Lindeberg 2016, Eq. 36)

100 $$\begin{aligned} \kappa _3 = \frac{2 (c+1) \sqrt{c^2-1} \, \tau ^{3/2}}{\left( c^2+c+1\right) }. \end{aligned}$$

Using the relationship between the centered and uncentered third-order moments

101 $$\begin{aligned} C_3&= \int \limits _{t = 0}^{\infty } (t - \delta )^3 \, h(t) \, \textrm{d}t \nonumber \\&= \int \limits _{t = 0}^{\infty } t^3 \, h(t) \, \textrm{d}t - 3 \delta \int _{t = 0}^{\infty } t^2 \, h(t) \, \textrm{d}t \nonumber \\&\quad + 3 \delta ^2 \int _{t = 0}^{\infty } t \, h(t) \, \textrm{d}t - 3 \delta ^3 \int _{t = 0}^{\infty } h(t) \, \textrm{d}t \nonumber \\&= M_3 - \frac{3 M_1}{M_0} M_2 + \frac{3 M_1^2}{M_0^2} M_1 - \frac{M_1^3}{M_0^3} M_0, \end{aligned}$$

we obtain the following expression for the normalized and centered third-order moment of the ex-Gaussian model

102 $$\begin{aligned} \kappa _3 = \frac{C_3}{M_0} = \frac{M_3}{M_0} - \frac{3 M_1 M_2}{M_0^2} + \frac{2 M_1^3}{M_0^3}. \end{aligned}$$

#### A.2.1 Method for third-order moment-based model fitting

To determine the parameters in the model based on the time-shifted time-causal limit kernel (98) with the DC-offset disregarded ($b_0 = 0$), we can hence proceed as follows:

1. Compute the unnormalized and uncentered moments $M_0$, $M_1$, $M_2$ and $M_3$ of the ex-Gaussian model according to (88), (89), (90) and (101).
2. Compute the variance *V* of the ex-Gaussian model according to (92) and let the variance $\tau$ of the time-causal limit kernel be equal to this value according to (94).
3. Identify the normalized and centered third-order moments of the ex-Gaussian model and the model based on the time-causal limit kernel according to (102) and (100).
4. With the third-order moment $\kappa _3$ of the ex-Gaussian model computed according to (102) and the variance $\tau$ of the time-causal limit kernel according to (94), square the expression (100) and solve the resulting fourth-order algebraic equation in terms of the distribution parameter *c* of the time-causal limit kernel. This will give four roots for *c*, out of which only two of the roots can be expected to satisfy the original unsquared equation, because of the squaring operation that may introduce new false roots.
5. Select^28^ the real root of the original Eq. (100) that additionally satisfies $c > 1$. Then, determine the temporal offset $t_0$ of the time-shifted time-causal limit kernel in (98) from the normalized and centered first-order moment of the time-causal limit kernel
  103 $$\begin{aligned} \delta = \sqrt{\frac{c+1}{c-1}} \sqrt{\tau } + t_0, \end{aligned}$$
  with $\delta$ identified with the normalized first-order moment of the ex-Gaussian model according to (91).
6. Compute the amplitude $b_1$ of the time-shifted time-causal limit kernel in (98) according to (95).

This procedure can either be carried out purely numerically or in a package for symbolic computation, such as Mathematica.

#### A.2.2 Experimental results

Figure 14 shows the result of applying this procedure for fitting a time-shifted time-causal limit kernel to an ex-Gaussian model that does not obey the assumptions for fitting a model based on the non-shifted time-causal limit kernel to it according to the previous second-order moment-based method. In the left figure, the result of the second-order moment-based method is shown, demonstrating a substantial difference because of the fixed zero offset of the original time-causal limit kernel. The right figure shows corresponding results for the third-order moment-based method, demonstrating a much better agreement between the two models, when an additional degree of flexibility is introduced into the model based on the time-causal limit kernel by adding the temporal offset parameter.

### A.3 Fitting models with the time-causal limit kernel to other functions or signals

Note that with replacement of the moments $M_0$, $M_1$, $M_2$ and optionally $M_3$ with the moments of some other non-negative function or signal, the same overall procedures can more generally be used for fitting models based on the time-causal limit kernel to other one-dimensional signals or functions that: (i) are defined for positive values of time, (ii) assume non-negative values only, (iii) have a roughly unimodal shape of first increasing and then decreasing and (iv) tend to zero towards infinity. The second-order moment-based fitting approach is in this context intended for situations when the temporal origin of the signal or function is known in advance and in some sense intended to be minimal, whereas the third-order moment-based fitting approach is intended for situations when the temporal origin in the data is unknown and hence needs to be adapted to each situation.

## B Appendix B: Implementing temporal filtering with a discrete approximation of the time-causal limit kernel

This appendix gives a brief explicit description about how to implement temporal filtering of a sampled discrete signal with a discrete approximation of the time-causal limit kernel.

For simplicity, assume^29^ that the input signal has been sampled with a unit time increment $\varDelta t = 1$. Then, given a temporal standard deviation of the kernel $\sigma$ in such units of time, compute its variance $\tau = \sigma ^2$ and choose a suitable value of the distribution parameter $c > 1$ that determines the sampling density in the temporal scale direction.

1. Compute a set of temporal scale levels $\tau _k$ according to a geometric distribution (12):
  104 $$\begin{aligned} \tau _k = c^{2(k-K)} \tau \quad \quad (1 \le k \le K). \end{aligned}$$
2. Compute a corresponding set of scale increments:
  105 $$\begin{aligned} \varDelta \tau _k = \tau _k - \tau _{k-1} \quad \quad (1 \le k \le K) \end{aligned}$$
  with the additional definition $\tau _0 = 0$.
3. Compute the time constants $\mu _k$ for a set of temporal recursive filters with generating functions of the form (54) according to (55):
  106 $$\begin{aligned} \mu _k = \frac{\sqrt{1 + 4 \varDelta \tau _k}-1}{2} \quad \quad (1 \le k \le K). \end{aligned}$$
4. Couple the following sets of first-order recursive filters in cascade (53):
  107 $$\begin{aligned} f_{\text{ out }}(t) - f_{\text{ out }}(t-1) = \frac{1}{1 + \mu _k} \, (f_{\text{ in }}(t) - f_{\text{ out }}(t-1)). \end{aligned}$$
  Note that, in a real-time scenario or an offline scenario where memory efficiency is important, if the task is to compute a single temporal scale level only, such as the first temporal scale level in a cascade, this operation can be performed without explicitly storing the representations at the intermediate temporal scale levels, except for at the current and the previous temporal frames.
  Furthermore, when computing multiple temporal scale levels in parallel, the temporal scale-space representation at the next coarser temporal scale is most efficiently computed by applying a single recursive filter to the temporal scale-space representation at the nearest finer temporal scale (if we assume a dense representation over temporal scale levels, where all the temporal scale levels are assumed to be used in the later processing stages).
5. Optionally, compute discrete approximations of scale-normalized temporal derivatives for some $\gamma > 0$ (where $\gamma = 1$ is a standard default value) by applying the following discrete derivative approximation operators (according to Eqs. 60 and 65)
  108 $$\begin{aligned} \delta _{\text{ t,norm }} = \sigma ^{\gamma } \, (1, -1) \quad \quad \delta _{\text{ tt,norm }} = \sigma ^{2 \gamma } \, (1, -2, 1) \end{aligned}$$
  to the temporally smoothed signal, alternatively instead using $L_p$-normalization according to (61) as opposed to variance-based normalization according to (60).

## Footnotes

1 For a popular overview over the wide range of temporal scales in physics and how the choice of temporal scale of observation thus will influence our modelling and understanding of the world, see ’t Hooft and Vandoren (2014).

2 Other names for this field, which develops methods for audio understanding by machines, are machine hearing (Lyon 2010, 2017) and computer audition.

3 For the reader interested in an overview of the developments of the different parts of temporal scale-space theory that this paper is based on, follows and extends, see the treatment in Sect. 9.

4 Note that in contrast to some other temporal scale-space formulations (Lindeberg 1997a, 2011; Fagerström 2005, 2007), we do not here assume a semi-group property over temporal scales, since such an assumption leads to poor temporal dynamics, *e.g.*, longer temporal delays given a variance-based measure of the temporal duration of the kernel, as explained in more detail in (Lindeberg 2017, Appendix 1).

5 In the general expression (3) for the bilateral Laplace-Stieltjes transform of a continuous scale-space kernel, the factor $e^{\gamma s^2}$ is the Laplace-Stieltjes transform of the Gaussian kernel $e^{-\gamma \xi ^2}$, the factor $1/(1 + a_i s)$ is the Laplace-Stieltjes transform of a truncated exponential function $e^{-a_i \xi }/a_i$ with time constant $a_i$, whereas the factors $e^{\delta s}$ and $e^{a_i s}$ correspond to translations in the temporal domain. Furthermore, the general product form of this expression in the Laplace-Stieltjes domain corresponds to a convolution of the corresponding primitives over the original temporal domain.

6 In certain literature, the property that we refer to as “covariance” is instead referred to as “equivariance”. In this paper, we throughout use the terminology “covariance”, to maintain consistency with the scale-space literature (Lindeberg 2013b).

7 A certain qualifier is, however, necessary in this context, since the ex-Gaussian model contains one more parameter than the model based on the time-causal limit kernel. Hence, the above mapping between these models is only valid if the temporal delay *m* in the ex-Gaussian model is not too large compared the temporal time constant $\mu$ and the amount of temporal smoothing $\sigma$. If a mapping is to be performed between the two models in the regime where this assumption does not hold, then an additional temporal delay parameter $t_0$ should be introduced into the model based on the time-causal limit kernel (98) at the cost of more complex analytical expressions for determining the parameters of the model based on the time-causal limit kernel from the temporal moments, now up to order 3, of the ex-Gaussian model, see Appendix A.2 for a further treatment of such an extension.

8 In the general expression (48) for the generating function of a discrete scale-space kernel, the factors $$1+\alpha _i z$$ and $$1+\delta _i z^{-1}$$ are the generating functions of generalized binomial filters of the form (49), the factors $$1-\beta _i z$$ and $$1-\gamma _i z^{-1}$$ are the generating functions of recursive filters of the form (50), the interpretation of the factor $$ e^{(q_{-1}z^{-1} + q_1z)}$$ is explained in Footnote 9, whereas the factor $$z^k$$ corresponds to a translation in the temporal domain. The product form of the overall expression in the domain of the generating functions does in turn correspond to convolutions of the corresponding primitives over the original temporal domain.

9 These kernels correspond to infinitely divisible distributions as can be described with the theory of Lévy processes (Sato 1999), where specifically the case $q_{-1} = q_1$ corresponds to convolution with the non-causal discrete analogue of the Gaussian kernel (Lindeberg 1990) and the case $q_{-1} = 0$ corresponds to convolution with time-causal temporal Poisson kernel (Lindeberg and Fagerström 1996; Lindeberg 1997a).

10 For notational convenience, and as is common in the field of scale-space theory, we write derivatives with subscripts, such that $L_t$ denotes the first-order derivative of the scale-space representation *L* with respect to time *t*, otherwise often written as $\frac{\partial L}{\partial t}$, and $L_{tt}$ denotes the second-order derivative, which can also be written as $\frac{\partial ^2 L}{\partial t^2}$. In a corresponding manner, $L_{t^n}$ denotes the *n*:th order derivative, elsewhere often written as $\frac{\partial ^n L}{\partial t^n}$. The operator $\partial _{t^n}$ denotes the *n*:th order temporal derivative operator, such that $L_{t^n} = \partial _{t^n} L$. The operator $\partial _{\zeta ^n}$ does in turn denote the *n*:th order scale-normalized temporal derivative operator, such that $L_{\zeta ^n} = \partial _{\zeta ^n} L$.

11 Here, using distribution parameter $c = 2$ for the time-causal limit kernel and scale normalization power $\gamma = 1$ for the scale-normalized temporal derivative operator.

12 Additionally, one usually states a requirement that the wavelet function should decrease sufficiently fast at the tails, such that $\int _{t=-\infty }^{\infty } (1 + |t|^{\alpha }) |\partial _{t^n} \varPsi | \, \textrm{d}t < \infty$ for some $\alpha > 0$. As will be shown later, for the temporal derivatives of the time-causal limit kernel, fulfilment of this condition follows from an exponential decrease towards zero at the infinity, see Eq. (75).

13 In wavelet analysis, the expansion of a test function onto a set of wavelet functions is usually computed in terms of inner products, corresponding to correlations. The reason why we instead use convolutions here is to avoid the additional step of reversing the time direction for the temporal derivatives of the time-causal limit kernel in relation to how they are used in the other parts of this article, in terms of convolutions.

14 In their work, Szu et al. (1992) propose optical computations to achieve real-time performance for their time-causal wavelets, whereas discrete approximations of the time-causal limit kernel can be expressed in terms of recursive filters, which in turn can be implemented in real time on standard digital signal processing hardware.

15 In this context, do specifically note that according to the classification of continuous scale-space kernels according to Sect. 2.1, the only possible continuous scale-space kernels are Gaussian kernels and truncated exponential kernels coupled in cascade. The Gaussian kernel is the canonical choice over a non-causal temporal domain, whereas composed convolutions of truncated exponential kernels are the only possible temporal scale-space kernels over a time-causal temporal domain. Out of the latter family of options, the time-causal limit kernel is a special choice that additionally allows for temporal scale covariance over a time-causal temporal domain, in a corresponding way as the regular Gaussian kernel allows for temporal scale covariance over a non-causal temporal domain.

16 Without taking the number of temporal scale levels *K* to infinity to enable true temporal scale covariance.

17 For a time-causal temporal filtering process that operates over multiple temporal scales, there will in general be a different temporal delay for each temporal scale, in the sense that the temporal delay will be shorter for temporal filtering over a short temporal scale and longer for temporal filtering over a long temporal scale. This raises an interesting theoretical problem concerning how to maintain an internally consistent representation of the time-delayed present, given that different components in such a representation may be associated with different temporal delays.

18 To understand how relative temporal ordering can be different for two observer with different relative velocities, consider two observers Alice and Bob in relation to a moving train with constant velocity. Alice is in a waggon of the train, whereas Bob is standing on the ground outside. Let us assume that Alice is positioned in the middle of the waggon, and uses a special flashlight to emit two photons simultaneously from exactly the middle of the waggon, one photon is emitted in the forward direction of the train and the other one is emitted in the backward direction. Since light always travels at the speed of light in relation to any Galilean frame according to Einstein, the two photons in the forward direction and the backward direction will hit the walls in the forward and the backward directions of the train simultaneously, from the viewpoint of Alice. From the viewpoint of Bob, the situation will, however, be different. Since the train moves in the forward direction, the photon emitted in forward direction will have to travel a longer distance from the temporal moment of emission to the temporal moment of arrival than the photon emitted in the backward direction, since the train is moving and changing the positions of the walls during the time it takes for the light to travel between the two positions. Therefore, the photon emitted in the forward direction will hit the wall in the forward direction after the photon emitted in the backward direction hits the wall in the backward direction. Thus, because of the way that space-time is transformed by high velocities, Alice and Bob will arrive at different conclusions regarding the relative temporal ordering of the two events.

19 A minor note concerning this thought experiment: If you find the situation artificial in the respect that the two photons arrive at the two walls exactly simultaneously from the viewpoint of Alice, you could modify the thought experiment slightly: Move the flashlight just a tiny bit in the forward direction, so that the photon emitted in the forward direction arrives at the forward wall slightly before the photon emitted in the backward wall from the viewpoint of Alice, but not too much so that that the photon emitted in the forward direction arrives at the forward wall before the photon emitted in the backward direction arrives at the backward wall from the viewpoint of Bob. Then, we have a complete reversal of the temporal ordering of the two temporal events.

20 For a biological perceptual agent, the relativistic time corrections that he or she may encounter due to relative velocities between two observers who observe everyday phenomena in the world with their own perceptual systems only will, however, be much shorter than the inner time scales of their perceptual systems, implying that relativistic time effects can be ignored in a treatment of how to handle the notion of time for a perceptual agent that observes everyday phenomena.

21 The view that the present is an instantaneous property does also go back to St. Augustine (Outler, transl. 1955, Book 11, page 194): “But the present has no extension whatever.”

22 In computational models of vision, such temporal changes can be measured in a *direct* way, by receptive fields in terms of temporal or spatio-temporal derivatives, in other words not by first perceiving the underlying spatial structures at each time moment and then inferring temporal relations as a secondary process, but in instead directly in the sense of using specific change detectors or motion detectors that operate directly on the spatio-temporal image structure caused by a dynamic scene.

23 Note, however, that this notion of an inner temporal scale for *any* representation of the present is, however, not the same notion as the notion of “the specious present” in the area of philosophy and psychology of time, a terminology forwarded by James (1890, pages 609–610), who stated that: “In short, the practically cognized present is no knife-edge, but a saddle-back, with a certain breadth of its own on which we sit perched, and from which we look in two directions into time. The unit of composition of our perception of time is a duration, with a bow and a stern, as it were—a rearward- and a forward-looking end. It is only as parts of this duration-block that the relation of succession of one end to the other is perceived.” A main difference between these concepts is that the notion of an inner temporal scale will be associated with any primitive that can be represented in the specious present, for example, in short-term temporal buffers of the immediate past, each with a different temporal delay.

24 Here, we disregard representations of the past that can be acquired by other external means, such as by communication with other individuals, by reading written records, by observing explicit video or audio recordings of the past, or by finding traces in the world of past events, as done in archeology or forensics.

25 The spatial scale selection methodology involves estimating the spatial size of image structures by detecting the spatial scale levels at which multi-scale spatial image operations assume their maxima over spatial scales (Lindeberg 1998a, 2021a).

26 Considering, for example, the sport of playing tennis. For a professional tennis player, the speed of the tennis ball after a serve may be up to the order 200 km/h, corresponding to more than 50 m/s. For among the faster temporal processes in human vision, the temporal limit of visual judgement is of the order of 20 ms, while for among some slower processes the limit of visual judgement is of the order of 100 ms (Holcombe 2009). Regarding spatio-temporal receptive fields in the primary visual cortex (V1), explicit modelling of examples of such receptive fields measured by DeAngelis et al. (1995), DeAngelis and Anzai (2004) lead to temporal scale values in the range from 50 to 80 ms (Lindeberg 2016). The composed temporal scale level for the entire visual hierarchy has to be longer, whereas the fastest possible visual reaction times are of the order of 200 ms (Jain et al. 2015). During temporal intervals in the range 20–100 ms, the tennis ball will be able to move by the order of 1–5 m, or during 200 ms even as far as 10 m (if we neglect the loss of speed because of the air resistance). As a tennis player, you quickly learn that you have to fixate on the ball in order to be able to hit it properly, which compensates for some amount of the relative motion. Nevertheless, it seems unlikely that we would be able to judge the position and the timing of the ball properly, unless our conscious perception of it involves at least some component of temporal extrapolation or prediction. Specifically, the process of fixating on a rapidly moving target also needs an explicit temporal prediction mechanism.

27 For further support of the working hypothesis that our perception of the present likely involves essential components of prediction or extrapolation in the forward direction of time, see Nijhawan (1994), Nijhawan (2008), Grush (2007), Grush (2008), Changizi et al. (2008) and White (2018).

## References

[CR1] Andoni S, Li N, Pollack GD (2007). Spectrotemporal receptive fields in the inferior colliculus revealing selectivity for spectral motion in conspecific vocalizations. J Neurosci.

[CR2] Atencio CA, Schreiner CE (2012). Spectrotemporal processing in spectral tuning modules of cat primary auditory cortex. PLoS ONE.

[CR3] Bright IM, Meister MLR, Cruzado NA, Tiganj Z, Buffalo EA, Howard MW (2020). A temporal record of the past with a spectrum of time constants in the monkey entorhinal cortex. Proc Natl Acad Sci.

[CR4] Buzsáki G, Llinás R (2017). Space and time in the brain. Science.

[CR5] Callender C (2017). What makes time special?.

[CR6] Cavanagh SE, Hunt LT, Kennerley SW (2020). A diversity of intrinsic timescales underlie neural computations. Front Neural Circuits.

[CR7] Chait M, Greenberg S, Arai T, Simon JZ, Poeppel D (2015). Multi-time resolution analysis of speech: evidence from psychophysics. Front Neurosci.

[CR8] Changizi MA, Hsieh A, Nijhawan R, Kanai R, Shimojo S (2008). Perceiving the present and a systematization of illusions. Cognit Sci.

[CR9] Chui CK (1992). An introduction to wavelets.

[CR10] Clark A (2016). Surfing uncertainty: prediction, action, and the embodied mind.

[CR11] Cohen L (1995). Time-frequency analysis.

[CR12] Conway BR, Kitaoka A, Yazdanbakhsh A, Pack CC, Livingstone MS (2005). Neural basis for a powerful static motion illusion. J Neurosci.

[CR13] Darwin C (2004). On the origin of species, 1859.

[CR14] Daubechies I (1992). Ten lectures on wavelets.

[CR15] DeAngelis GC, Anzai A, Chalupa LM, Werner JS (2004). A modern view of the classical receptive field: linear and non-linear spatio-temporal processing by V1 neurons. The visual neurosciences.

[CR16] DeAngelis GC, Ohzawa I, Freeman RD (1995). Receptive field dynamics in the central visual pathways. Trends Neurosci.

[CR17] Debnath L, Shah FA (2002). Wavelet transforms and their applications.

[CR18] den Brinker AC, Roufs JAJ (1992). Evidence for a generalized Laguerre transform of temporal events by the visual system. Biol Cybern.

[CR19] Einstein A (1905) Zur Elektrodynamik bewegter Körper. Annalen der Physik 4

[CR20] Einstein A (1916) Relativity: the special and general theory. Methuen & Co, Ltd. Translated by R. W. Lawson. https://gutenberg.org/ebooks/5001

[CR21] Elhilali M, Fritz J, Chi T-S, Shamma S (2007). Auditory cortical receptive fields: stable entities with plastic abilities. J Neurosci.

[CR22] Fagerström D (2005). Temporal scale-spaces. Int J Comput Vis.

[CR23] Fagerström D (2007) Spatio-temporal scale-spaces. In: Gallari F, Murli A, Paragios N (eds) Proceedings of international conference on scale-space theories and variational methods in computer vision (SSVM 2007), volume 4485 of Springer LNCS. Springer, pp 326–337

[CR24] Feichtinger HG, Gröchenig K (1992) Gabor wavelets and the Heisenberg group: Gabor expansions and short time Fourier transform from the group theoretical point of view. In: Chui CK (ed) Wavelets: a tutorial in theory and applications, volume 2. Academic Press, pp 359–398

[CR25] Feichtinger HG, Strohmer T (1998). Gabor analysis and algorithms: theory and applications.

[CR26] Flandrin P (2018). Explorations in time-frequency analysis.

[CR27] Florack LMJ (1997). Image structure. Series in mathematical imaging and vision.

[CR28] Friston K (2010). The free-energy principle: a unified brain theory?. Nat Rev Neurosci.

[CR29] Gabor D (1946). Theory of communication. J IEE.

[CR30] Gauthier B, Eger E, Hesselmann G, Giraud A-L, Kleinschmidt A (2012). Temporal tuning properties along the human ventral visual stream. J Neurosci.

[CR31] Gentner TQ (2008). Temporal scales of auditory objects underlying birdsong vocal recognition. J Acoust Soc Am.

[CR32] Goldman MS (2009). Memory without feedback in a neural network. Neuron.

[CR33] Graps A (1995). An introduction to wavelets. IEEE Comput Sci Eng.

[CR34] Gröchenig K (2001). Foundations of time-frequency analysis.

[CR35] Grossmann A, Morlet J (1984). Decomposition of Hardy functions into square integrable wavelets of constant shape. SIAM J Math Anal.

[CR36] Grush R (2007) Time and experience. In: Müller T (ed) Philosophie der Zeit. Klostermann, pp 27–44

[CR37] Grush R (2008). Temporal representation and dynamics. New Ideas Psychol.

[CR38] Grushka E (1972). Characterization of exponentially modified Gaussian peaks in chromatography. Anal Chem.

[CR39] Guo C, Huson V, Macosko EZ, Regehr WG (2021). Graded heterogeneity of metabotropic signaling underlies a continuum of cell-intrinsic temporal responses in unipolar brush cells. Nat Commun.

[CR40] Gütig R, Sompolinsky H (2006). The tempotron: a neuron that learns spike timing-based decisions. Nat Neurosci.

[CR41] Heil CE, Walnut DF (1989). Continuous and discrete wavelet transforms. SIAM Rev.

[CR42] Hewitt MJ, Meddis R (1994). A computer model of amplitude-modulation sensitivity of single units in the inferior colliculus. J Acoust Soc Am.

[CR43] Holcombe AO (2009). Seeing slow and seeing fast: two limits on perception. Trends Cogn Sci.

[CR44] Hosken JWJ (1988). Ricker wavelets in their various guises. First Break.

[CR45] Howard MW (2021) Memory for time. In: Oxford handbook of human memory. Oxford University Press. submitted

[CR46] Howard MW, Hasselmo ME (2020) Cognitive computation using neural representations of time and space in the Laplace domain. arXiv:2003.11668

[CR47] Howard MW, Luzardo A, Tiganj Z (2018). Evidence accumulation in a Laplace domain decision space. Comput Brain Behav.

[CR48] Iijima T (1962). Basic theory on normalization of pattern (in case of typical one-dimensional pattern). Bull Electrotechn Lab.

[CR49] Jain A, Bansal R, Kumar A, Singh KD (2015). A comparative study of visual and auditory reaction times on the basis of gender and physical activity levels of medical first year students. Int J Appl Basic Med Res.

[CR50] James W (1890). The principles of psychology.

[CR51] Jansson Y, Lindeberg T (2018). Dynamic texture recognition using time-causal and time-recursive spatio-temporal receptive fields. J Math Imag Vis.

[CR52] Johannesma PIM (1972) The pre-response stimulus ensemble of neurons in the cochlear nucleus. In: IPO symposium on hearing theory. Eindhoven, The Netherlands, pp 58–69

[CR53] Kant I (1902) Prolegomena to any future metaphysics (Prolegomena zu einer jeden künftigen Metaphysik, die als Wissenschaft wird auftreten können 1783). Open Court. Translated by Paul Carus

[CR54] Karlin S (1968). Total positivity.

[CR55] Koch C (1999). Biophysics of computation: information processing in single neurons.

[CR56] Koenderink JJ (1984). The structure of images. Biol Cybern.

[CR57] Koenderink JJ (1988). Scale-time. Biol Cybern.

[CR58] Koenderink JJ, Albertazzi L, Thonder V, Gert J, Vishwanath D (2011). Vision and information. Perception beyond inference: the information content of visual processes.

[CR59] Koenderink JJ, van Doorn AJ (1987). Representation of local geometry in the visual system. Biol Cybern.

[CR60] Koenderink JJ, van Doorn AJ (1992). Generic neighborhood operators. IEEE Trans Pattern Anal Mach Intell.

[CR61] Kwon Y-H, Park M-G (2019) Predicting future frames using retrospective cycle GAN. In: Proceedings of computer vision and pattern recognition (CVPR 2019), pp 1811–1820

[CR62] Latimer KW, Barbera D, Sokoletsky M, Awwad B, Katz Y, Nelken I, Lampl I, Fairhall AL, Priebe NJ (2019). Multiple timescales account for adaptive responses across sensory cortices. J Neurosci.

[CR63] Lindeberg T (1990). Scale-space for discrete signals. IEEE Trans Pattern Anal Mach Intell.

[CR64] Lindeberg T (1993). Effective scale: a natural unit for measuring scale-space lifetime. IEEE Trans Pattern Anal Mach Intell.

[CR65] Lindeberg T (1993). Scale-space theory in computer vision.

[CR66] Lindeberg T (1994). Scale-space theory: a basic tool for analysing structures at different scales. J Appl Stat.

[CR67] Lindeberg T (1997) On automatic selection of temporal scales in time-casual scale-space. In: Sommer G, Koenderink JJ (eds) Proc. AFPAC’97: algebraic frames for the perception-action cycle, volume 1315 of Springer LNCS. Kiel, Germany, pp 94–113

[CR68] Lindeberg T (1997b) Linear spatio-temporal scale-space. In: Proceedings of international conference on scale-space theory in computer vision (Scale-Space’97), volume 1252 of Springer LNCS. Springer, pp 113–127

[CR69] Lindeberg T (1998). Feature detection with automatic scale selection. Int J Comput Vis.

[CR70] Lindeberg T (1998). Edge detection and ridge detection with automatic scale selection. Int J Comput Vis.

[CR71] Lindeberg T (2001) Linear spatio-temporal scale-space. Technical Report ISRN KTH/NA/P–01/22–SE, Dept. of Numerical Analysis and Computer Science, KTH, Nov. http://www.csc.kth.se/cvap/abstracts/cvap257.html

[CR72] Lindeberg T (2011). Generalized Gaussian scale-space axiomatics comprising linear scale-space, affine scale-space and spatio-temporal scale-space. J Math Imag Vis.

[CR73] Lindeberg T (2013). A computational theory of visual receptive fields. Biol Cybern.

[CR74] Lindeberg T, Hawkes P (2013). Generalized axiomatic scale-space theory. Advances in imaging and electron physics.

[CR75] Lindeberg T (2015) Separable time-causal and time-recursive spatio-temporal receptive fields. In: Proceedings of scale space and variational methods in computer vision (SSVM 2015), volume 9087 of Springer LNCS, pp 90–102

[CR76] Lindeberg T (2016). Time-causal and time-recursive spatio-temporal receptive fields. J Math Imag Vis.

[CR77] Lindeberg T (2017). Temporal scale selection in time-causal scale space. J Math Imag Vis.

[CR78] Lindeberg T (2018). Spatio-temporal scale selection in video data. J Math Imag Vis.

[CR79] Lindeberg T (2018). Dense scale selection over space, time and space-time. SIAM J Imag Sci.

[CR80] Lindeberg T, Ikeuchi K (2021). Scale selection. Computer vision.

[CR81] Lindeberg T (2021). Normative theory of visual receptive fields. Heliyon.

[CR82] Lindeberg T, Fagerström D (1996) Scale-space with causal time direction. In: Proceedings of European conference on computer vision (ECCV’96), volume 1064 of Springer LNCS. Cambridge, UK, pp 229–240

[CR83] Lindeberg T, Friberg A (2015). Idealized computational models of auditory receptive fields. PLoS ONE.

[CR84] Lindeberg T, Friberg A (2015b) Scale-space theory for auditory signals. In: Proceedings of scale space and variational methods in computer vision (SSVM 2015), volume 9087 of Springer LNCS, pp 3–15

[CR85] Lotter W, Kreiman G, Cox D (2020). A neural network trained to predict future video frames mimics critical properties of biological neuronal responses and perception. Nat Mach Intell.

[CR86] Luczak A, McNaughton BL, Kubo Y (2022). Neurons learn by predicting future activity. Nat Mach Intell.

[CR87] Lyon RF (2010). Machine hearing: an emerging field. IEEE Signal Process Mag.

[CR88] Lyon RF (2017). Human and machine hearing: extracting meaning from sound.

[CR89] Machens CK, Wehr MS, Zador AM (2004). Linearity of cortical receptive fields measures with natural sounds. J Neurosci.

[CR90] Mallat SG (1989). A theory for multiresolution signal decomposition: the wavelet representation. IEEE Trans Pattern Anal Mach Intell.

[CR91] Mallat SG (1999). A wavelet tour of signal processing.

[CR92] Marr D (1982). Vision: a computational investigation into the human representation and processing of visual information.

[CR93] Marr DC (1976). Early processing of visual information. Philos Trans R Soc (B).

[CR94] McCrone J (2022). Friston’s theory of everything. Lancet Neurol.

[CR95] McTaggart JE (1908) The unreality of time. Mind, vol 17, no 68, pp 457–474

[CR96] Meyer Y (1992). Wavelets and operators.

[CR97] Miri A, Bhasin BJ, Aksay ERF, Tank DW, Goldman MS (2022). Oculomotor plant and neural dynamics suggest gaze control requires integration on distributed timescales. J Physiol.

[CR98] Mölder B, Arstila V, Øhrstrøm P (2016). Philosophy and psychology of time.

[CR99] Monsa R, Peer M, Arzy S (2020). Processing of different temporal scales in the human brain. J Cognit Neurosci.

[CR100] Nijhawan R (1994). Motion extrapolation in catching. Nature.

[CR101] Nijhawan R (2008). Visual prediction: psychophysics and neurophysiology of compensation for time delays. Behav Brain Sci.

[CR102] Osman AF, Lee CM, Escabí MA, Read HL (2018). A hierarchy of time scales for discriminating and classifying the temporal shape of sound in three auditory cortical fields. J Neurosci.

[CR103] Outler AC (1955) St. Augustine: confessions. Grand rapids, MI: Christian Classics Ethereal Library. https://www.ccel.org/ccel/augustine/confess.html

[CR104] Paolucci C (2021) Perception as controlled hallucination. In: Cognitive semiotics, volume 24. Springer, pp 127–157

[CR105] Patterson RD, Nimmo-Smith I, Holdsworth J, Rice P (1987) An efficient auditory filterbank based on the Gammatone function. In: A meeting of the IOC Speech Group on Auditory Modelling at RSRE 2:7

[CR106] Patterson RD, Allerhand MH, Giguere C (1995). Time-domain modeling of peripheral auditory processing: a modular architecture and a software platform. J Acoust Soc Am.

[CR107] Power SE (2016) Relative and absolute temporal presence. In: Philosophy and psychology of time. Springer, pp 69–100

[CR108] Qian S, Chen D (1999). Joint time-frequency analysis. IEEE Signal Process Mag.

[CR109] Qiu A, Schreiner CE, Escabi MA (2003). Gabor analysis of auditory midbrain receptive fields: spectro-temporal and binaural composition. J Neurophysiol.

[CR110] Ricker N (1944). Wavelet functions and their polynomials. Geophysics.

[CR111] Rioul O, Duhamel P (1992). Fast algorithms for discrete and continuous wavelet transforms. IEEE Trans Inf Theory.

[CR112] Rivero-Moreno CJ, Bres S (2004) Spatio-temporal primitive extraction using Hermite and Laguerre filters for early vision video indexing. In: Image analysis and recognition, volume 3211 of Springer LNCS, pp 825–832

[CR113] Sato K-I (1999). Lévy processes and infinitely divisible distributions. Cambridge studies in advanced mathematics.

[CR114] Schoenberg IJ (1930). Über variationsvermindernde lineare transformationen. Math Z.

[CR115] Schoenberg IJ (1946). Contributions to the problem of approximation of equidistant data by analytic functions. Q Appl Math.

[CR116] Schoenberg IJ (1947). On totally positive functions, Laplace integrals and entire functions of the Laguerre–Pòlya–Schur type. Proc Natl Acad Sci.

[CR117] Schoenberg IJ (1948) Some analytical aspects of the problem of smoothing. In: Courant anniversary volume, studies and essays. New York, pp 351–370

[CR118] Schoenberg IJ (1950). On Pòlya frequency functions. ii. Variation-diminishing integral operators of the convolution type. Acta Sci Math (Szeged).

[CR119] Schoenberg IJ (1953). On smoothing operations and their generating functions. Bull. Am. Math. Soc..

[CR120] Schoenberg IJ (1988) I. J. schoenberg selected papers, volume 2. Springer. Edited by C. de Boor

[CR121] Seth A (2021). Being you: a new science of consciousness.

[CR122] Singer Y, Teramoto Y, Willmore BDB, Schnupp JWH, King AJ, Harper NS (2018). Sensory cortex is optimized for prediction of future input. Elife.

[CR123] Spencer H (2020) The principles of biology: volume 1, 1864. Outlook Verlag

[CR124] Spitmaan M, Seo H, Lee D, Soltani A (2020). Multiple timescales of neural dynamics and integration of task-relevant signals across cortex. Proc Natl Acad Sci.

[CR125] Sporring J, Nielsen M, Florack L, Johansen P (eds) (1997) Gaussian scale-space theory: proceedings of PhD school on scale-space theory. Series in mathematical imaging and vision. Springer, Copenhagen, Denmark

[CR126] Szu HH, Telfer BA, Lohmann AW (1992) Causal analytical wavelet transform. Opt Eng 31(9):1825–1829

[CR127] ’t Hooft G, Vandoren S (2014). Time in powers of ten: natural phenomena and their timescales.

[CR128] Teng X, Tian X, Poeppel D (2016). Testing multi-scale processing in the auditory system. Sci Rep.

[CR129] ter Haar Romeny B (2003). Front-end vision and multi-scale image analysis.

[CR130] ter Haar Romeny B, Florack L, Nielsen M (2001) Scale-time kernels and models. In: Proceedings of international conference on scale-space and morphology in computer vision (Scale-Space’01), volume 2106 of Springer LNCS. Vancouver, Canada

[CR131] Tsao A, Sugar J, Lu L, Wang C, Knierim JJ, Moser M-B, Moser EI (2018). Integrating time from experience in the lateral entorhinal cortex. Nature.

[CR132] van der Berg ES, Reyneke PV, de Ridder C (2014) Rotational image correlation in the Gauss-Laguerre domain. In: Third SPIE conference on sensors, MEMS and electro-optic systems: proceedings of SPIE, volume 9257, pp 92570F–1–92570F–17

[CR133] Weickert J, Ishikawa S, Imiya A (1999). Linear scale-space has first been proposed in Japan. J Math Imag Vis.

[CR134] White PA (2018). Is the perceived present a predictive model of the objective present?. Vis Cognit.

[CR135] White PA (2020). The perceived present: what is it, and what is it there for?. Psychon Bull Rev.

[CR136] Witkin AP (1983) Scale-space filtering. In: Proceedings of 8th international joint conference art. Intell. Karlsruhe, Germany, pp 1019–1022

[CR137] Wittmann M (2009). The inner experience of time. Philos Trans R Soc B Biol Sci.

[CR138] Wohlgemuth A (1911) On the after-effect of seen movement. Br J Psychol Monogr Suppl, pp 1–117

[CR139] Zalta EN (2020) Time. In Stanford encyclopedia of philosophy. Metaphysics Research Lab, Philosophy Department, Stanford University. https://plato.stanford.edu/entries/time/

